# cGAS Downregulation Contributes to EGFR-TKI Resistance in NSCLC through the p-Nrf2–SIRT3–ROS/Ferroptosis Axis

**DOI:** 10.32604/or.2026.082400

**Published:** 2026-09-14

**Authors:** Yawan Zi, Huilin Yu, Xiaohui Wang, Yuezhou Zhang, Shengxin Fan, Jiukang Li, Jian Wang, Ke Liao, Hong Chen

**Affiliations:** 1 Department of Pulmonary and Critical Care Medicine, The First Affiliated Hospital of Chongqing Medical University, Chongqing, China; 2 Department of Infectious Diseases, The People’s Hospital of Yue Chi County, Guang’an, China; 3 Emergency and Intensive Care Medicine Center, Guang’an People’s Hospital, Guang’an, China

**Keywords:** Cyclic GMP-AMP synthase, ferroptosis, EGFR-TKI resistance, p-Nrf2-SIRT3-ROS axis, DNA damage repair

## Abstract

**Background:** Although epidermal growth factor receptor (EGFR)-directed tyrosine kinase inhibition produces substantial initial benefit in EGFR-mutant non-small cell lung cancer, durable disease control is frequently compromised by the emergence of drug-resistant tumor cells. We therefore examined whether loss of cyclic guanosine monophosphate-adenosine monophosphate synthase (cGAS) supports the resistant phenotype by altering redox control and the cellular threshold for ferroptotic injury. **Methods:** The Gene Expression Omnibus (GEO) datasets GSE172002 and GSE236654 were analyzed to identify resistance-associated pathways. cGAS was depleted in parental cells and restored in resistant derivatives, followed by phenotypic, redox, mitochondrial, and signaling assessments in cultured cells and xenografts; pathway relationships were further examined by rescue experiments and structural modeling. **Results:** Bioinformatics analysis indicated significant alteration of DNA repair-related pathways in epidermal growth factor receptor tyrosine kinase inhibitor (EGFR-TKI)-resistant models. Resistant PC-9/GR and H1975/OR cells displayed increased half-maximal inhibitory concentration (IC_50_) values and attenuated inhibition of phosphorylated EGFR (p-EGFR), Phosphorylated Protein Kinase B1 (p-AKT1), and phosphorylated extracellular signal-regulated kinase 1/2 (p-ERK1/2) after matched EGFR-TKI treatment. In parental cells, EGFR-TKI exposure was associated with increased DNA damage, cytosolic double-stranded DNA (dsDNA) accumulation, cGAS induction, ferroptosis-related staining patterns, reduced glutathione (GSH), and increased malondialdehyde (MDA), whereas these changes were less evident in resistant cells and were partly attenuated by Ferrostatin-1. Functionally, cGAS knockdown was associated with enhanced proliferative, migratory/invasive, and xenograft growth phenotypes, together with a redox pattern consistent with reduced ferroptosis susceptibility. Conversely, cGAS overexpression in resistant cells produced opposite effects. Altering cGAS abundance redistributed total and Ser40-phosphorylated nuclear factor erythroid 2–related factor 2 (Nrf2) between cellular compartments and concurrently changed sirtuin 3 (SIRT3) abundance and deacetylase activity. Nrf2 or SIRT3 manipulation partially reversed cGAS-associated phenotypes. **Conclusions:** These findings identify low cGAS abundance as a feature of the resistant state and support its involvement in an Nrf2–SIRT3-dependent antioxidant program that raises the threshold for ferroptotic damage.

## 1 Introduction

The therapeutic landscape of EGFR-mutant lung cancer changed substantially after the introduction of epidermal growth factor receptor tyrosine kinase inhibitors (EGFR-TKIs) [1]. Across successive drug generations, agents such as gefitinib, afatinib, and osimertinib inhibit mutant EGFR signaling and delay disease progression in lung adenocarcinoma carrying activating EGFR alterations [2]. Nevertheless, the clinical benefit is rarely permanent because tumor cells eventually escape EGFR inhibition. Tumors can evade EGFR blockade through several biologically distinct routes, ranging from additional EGFR mutations to bypass-pathway reactivation and changes in histological identity [3]. Although osimertinib can overcome T790M-mediated resistance, secondary alterations such as C797S mutation and reactivation of bypass signaling may contribute to subsequent resistance [3]. These observations indicate that EGFR-TKI resistance is biologically heterogeneous, and additional adaptive mechanisms remain to be clarified.

DNA damage response (DDR) programs have been increasingly linked to EGFR-TKI response and resistance [4]. Pharmacological blockade of EGFR may produce replication-associated stress and DNA double-strand lesions, thereby recruiting homologous recombination and non-homologous end-joining machinery [5]. Resistant populations may withstand continued drug exposure by reconfiguring repair machinery, including ATM, PARP1, and factors participating in homologous recombination or non-homologous end joining [5]. Inhibition of EGFR signaling can also promote chromosomal instability and mutation accumulation, thereby facilitating resistance-associated events such as T790M mutation or MET amplification [3,6]. Although EGFR-TKI exposure has been associated with compensatory activation of DDR-related programs [7], the precise relationship between DNA repair-associated changes, cytosolic DNA stress, and EGFR-TKI resistance remains incompletely defined.

Following DNA damage, unrepaired chromosomal fragments or micronuclear DNA may become exposed to the cytoplasm during cell division [8]. Cyclic GMP-AMP synthase (cGAS) is a cytosolic DNA sensor that recognizes aberrant double-stranded DNA and can activate STING-dependent innate immune signaling [9,10]. Through this function, cGAS connects genome instability with inflammatory responses, antitumor immunity, and cellular stress adaptation [11]. In cancer, however, the biological effects of cGAS signaling are context-dependent and may vary according to tumor type, treatment pressure, and microenvironmental conditions. Although the canonical cGAS-STING pathway has been widely studied, the tumor cell-autonomous role of cGAS in EGFR-TKI-resistant NSCLC has not been fully characterized.

Emerging evidence also suggests that cGAS may be linked to oxidative stress, mitochondrial function, and lipid metabolism [12,13]. Mitochondrial damage and mitochondrial DNA stress can activate cGAS–STING signaling [14,15]. Conversely, mitochondria-localized cGAS has been reported to suppress ferroptosis in hepatocellular carcinoma cells, indicating that the relationship between cGAS, mitochondrial homeostasis, and ferroptosis may be context-dependent [16]. It may also influence lipid peroxidation-related processes through pathways involving acyl-CoA synthetase long-chain family member 4 (ACSL4) and polyunsaturated fatty acid metabolism [17]. Ferroptotic injury arises when iron-catalyzed oxidation of membrane phospholipids exceeds the capacity of glutathione-dependent and related antioxidant systems [18]. In lung cancer, ferroptosis susceptibility has been implicated in tumor progression and therapeutic response [19]. One adaptive route available to resistant cells is reinforcement of peroxide-detoxifying capacity, for example by increasing GPX4 or SLC7A11/xCT activity and thereby preserving the intracellular glutathione pool [20]. These observations suggest that ferroptosis-related redox adaptation may contribute to the survival of EGFR-TKI-resistant cells.

The Nrf2/SIRT3 antioxidant program may represent an additional layer of this adaptive response. Nrf2 is a key transcriptional regulator of antioxidant and stress-response genes [21,22], whereas SIRT3 contributes to mitochondrial redox balance and metabolic homeostasis [23]. Nrf2 has been reported to bind the SIRT3 promoter and regulate SIRT3 transcription [24]. In the setting of EGFR-TKI resistance, increased Nrf2/SIRT3-associated antioxidant capacity may help tumor cells limit ROS accumulation and ferroptosis-related injury. However, whether altered cGAS expression is connected to Nrf2/SIRT3 signaling and ferroptosis susceptibility in EGFR-TKI-resistant NSCLC remains unclear.

It is not yet known whether the decline in cGAS observed during acquisition of EGFR-TKI resistance is functionally connected to the ferroptosis-defensive state of resistant NSCLC cells. In particular, it remains unclear whether cGAS downregulation contributes to the adaptive survival state of EGFR-TKI-resistant NSCLC cells, and whether this process is associated with altered DNA damage-related stress responses, Nrf2/SIRT3-mediated redox regulation, and ferroptosis-related vulnerability. We addressed this question by comparing parental and resistant models and by testing whether cGAS abundance controls redox and ferroptotic phenotypes through Nrf2 phosphorylation and SIRT3-dependent mitochondrial regulation. By focusing on this treatment-resistant context, we aimed to clarify a disease- and therapy-relevant role of cGAS beyond its broader functions in innate immune signaling.

## 2 Materials and Methods

### 2.1 Bioinformatics Analysis

Two Gene Expression Omnibus (GEO) transcriptomic datasets (https://www.ncbi.nlm.nih.gov/geo/), GSE172002 and GSE236654, containing EGFR-TKI-sensitive and -resistant non-small cell lung cancer (NSCLC) cell models, were analyzed. For each accession, the available count-level or normalized expression data were retrieved together with the platform annotation. Group labels were reconstructed directly from the sample descriptions supplied by the original submitters.

Expression differences between resistant and sensitive cells were evaluated with the limma package (version 3.66.0) in R (version 4.5.1; R Foundation for Statistical Computing, Vienna, Austria). Within each dataset, genes satisfying both |log_2_ fold change| ≥ 1 and *p* < 0.05 were classified as differentially expressed genes (DEGs). When multiple-testing correction was required, the Benjamini–Hochberg procedure was applied. Direction-consistent overlapping DEGs between datasets were visualized using the ggVennDiagram package (version 1.5.7). Gene set enrichment analysis (GSEA) was performed in R software (version 4.5.1) using the clusterProfiler package (version 4.18.4). The Molecular Signatures Database (MSigDB) Hallmark gene sets were obtained using the msigdbr package (version 26.1.0). Genes were ranked according to signed differential statistics, and enrichment was interpreted using normalized enrichment score (NES) and false discovery rate (FDR) q value. The GSEA results for the HALLMARK_DNA_REPAIR pathway in the EGFR-TKI resistance datasets are summarized in Table 1. The WikiPathways Cytosolic DNA-sensing pathway gene set (WP4655) was intersected with overlapping DEGs to identify candidate genes potentially related to cytosolic DNA sensing and EGFR-TKI resistance.

To assess the potential clinical significance of the identified candidate genes, their expression profiles in lung adenocarcinoma (LUAD) and lung squamous cell carcinoma (LUSC) were examined through the Gene Set Cancer Analysis platform (GSCA; https://guolab.wchscu.cn/GSCA/). The prognostic association of each candidate was subsequently investigated using the Kaplan–Meier Plotter database (https://kmplot.com/analysis/). Patients were divided into high- and low-expression cohorts on the basis of the cutoff automatically defined by the database, and differences in overall survival between the two cohorts were evaluated with the log-rank test.

**Table 1:** GSEA of the hallmark DNA repair pathway in EGFR-TKI resistance datasets.

GSE	ID	Set Size	Enrichment Score	NES	*p* Value	Adjusted *p* Value (FDR)	q Value	Core Enrichment
GSE172002	HALLMARK_DNA_REPAIR	148	0.320542662945513	1.40716169286129	0.00856547962537867	0.0178447492195389	0.0086406154115662	TYMS/DDB1/SF3A3/SAC3D1/NELFCD/GTF2H1/ELOA/POLR2E/POLR1C/NME1/SSRP1/FEN1/POLA2/DGCR8/RAE1/RFC3/CLP1/GTF2A2/SMAD5/ITPA/RPA2/ERCC2/PRIM1/IMPDH2/POLR2I/RFC4/GSDME/POLA1/GTF2H3/TAF9/EIF1B/POLD3/POLR2G/PCNA/STX3/RFC5/RAD51/RAD52/REV3L/RALA/RNMT/NUDT21/DUT/CCNO/TMED2/SNAPC4/ALYREF/NT5C/SDCBP
GSE236654	HALLMARK_DNA_REPAIR	148	−0.42913030937063	−1.7276419662203	0.0000456145937365924	0.000190060807235802	0.0000680217625896553	GTF2A2/GMPR2/POLE4/GTF2B/CSTF3/GTF2H3/POLL/ERCC8/RAD51/GTF3C5/CANT1/POLD3/TMED2/AK1/TSG101/RPA2/USP11/ERCC3/CETN2/DUT/DDB1/DGCR8/NELFCD/TYMS/MRPL40/NT5C3A/CCNO/NELFB/POLA2/EDF1/SEC61A1/RFC4/BCAP31/CMPK2/POLR2K/POLR2E/POLR2D/BOLA2/RFC3/POLR2A/PRIM1/POLR2C/POLR2F/GTF2F1/DCTN4/POLR1C/ITPA/RAE1/CLP1/RALA/SSRP1/ELOA/NT5C/APRT/STX3/RFC2/SAC3D1/ARL6IP1/LIG1/UMPS/POLA1/TARBP2/IMPDH2/SF3A3/ZWINT/ALYREF/PCNA/RPA3/TAF9/SUPT4H1/ADRM1/FEN1/PNP/NME1

Abbreviations: DNA, deoxyribonucleic acid; EGFR-TKI, epidermal growth factor receptor tyrosine kinase inhibitor; FDR, false discovery rate; GSE, Gene Expression Omnibus Series; GSEA, gene set enrichment analysis; ID, identifier; NES, normalized enrichment score. Gene names listed in the Core Enrichment column are presented using the official gene symbols approved by the HUGO Gene Nomenclature Committee (HGNC).

### 2.2 Molecular Docking Workflow

Protein docking was undertaken to generate testable structural models of a possible cGAS–Nrf2 contact surface. The predicted Nrf2 structure was retrieved from the AlphaFold Protein Structure Database (AlphaFold DB; developed by Google DeepMind and EMBL-EBI; https://alphafold.ebi.ac.uk) using UniProt accession Q16236. Model confidence was inspected in PyMOL Molecular Graphics System (version 2.5.4; Schrödinger, LLC, New York, NY, USA; https://pymol.org) by displaying the predicted local distance difference test (pLDDT) values stored in the B-factor field. Only residues with pLDDT values greater than 90 were included in the high-confidence subset. The cap ‘n’ collar basic leucine zipper (CNC-bZIP) domain of Nrf2, spanning residues 457–567, was then isolated for docking, with the native residue numbering retained.

The experimentally resolved cGAS structure was downloaded from the RCSB Protein Data Bank (RCSB PDB; https://www.rcsb.org; PDB ID: 9MDC). Chain A was selected, whereas crystallographic water molecules, bound ligands, and non-protein atoms were excluded before analysis. Initial rigid-body docking was conducted in Balanced mode using the ClusPro 2.0 server (Vajda Lab and ABC Group, Boston University and Stony Brook University; https://cluspro.org), and the centers of the highest-ranked clusters were carried forward as starting conformations. These preliminary complexes were further refined with the HADDOCK 2.4 web server (Computational Structural Biology Group, Utrecht University; https://wenmr.science.uu.nl/haddock2.4/). Ambiguous interaction restraints were assigned according to previously reported functional regions and residues with a relative solvent accessibility of at least 15%. The active residues were defined as Nrf2 residues 479, 481–485, 499–503, 505–506, and 508–509, together with cGAS residues 211, 225, 227, 239–240, 319, 347, 353, 362, 376, and 436–437 [25,26]. Passive residues were automatically assigned by HADDOCK within 6.5 Å. Docking models were ranked according to HADDOCK score, cluster size, and interface root-mean-square deviation.

This docking analysis was performed using a structural model of total Nrf2 and the extracted CNC-bZIP region. It was intended to provide a computational hypothesis for a possible cGAS–Nrf2 interface, rather than a phospho-Ser40-specific cGAS–p-Nrf2 model. Because Ser40 is located in the N-terminal region and was not included in the docked fragment, the docking results were interpreted cautiously and were not considered direct structural evidence for a phosphorylation-state-specific interaction.

### 2.3 Cell Culture and Establishment of EGFR-TKI-Resistant Cell Models

Human NSCLC cell lines PC-9 (Cat. No. YS7836C) and its gefitinib-resistant derivative PC-9/GR (Cat. No. YS2906C) were purchased from Shanghai Yaji Biotechnology Co., Ltd. (Shanghai, China). H1975 (Cat. No. CTCC-001-0354-CM) and its osimertinib-resistant derivative H1975/OR (Cat. No. CTCC-0281-NY) were purchased from Zhejiang Meisen Cell Technology Co., Ltd. (Zhejiang, China). The suppliers confirmed the identity of all cell lines by short tandem repeat (STR) profiling. Experiments were conducted using cultures within a restricted passage range after thawing. Mycoplasma screening was performed routinely, and only cultures with negative test results were used.

PC-9 and PC-9/GR cells were propagated in Dulbecco’s modified Eagle medium (DMEM; Gibco, Thermo Fisher Scientific, New York, NY, USA; Cat. No. 31870074). H1975 and H1975/OR cells were grown in RPMI-1640 medium obtained from Gibco (Cat. No. 31870082). Both basal media were supplemented with 10% fetal bovine serum (FBS; PAN-Biotech, Aidenbach, Germany; Cat. No. P30-3302) and 1% penicillin–streptomycin solution (100×; Beyotime Biotechnology, Shanghai, China; Cat. No. C0222), yielding final concentrations of 100 U/mL penicillin and 100 μg/mL streptomycin. All cultures were maintained at 37°C in a humidified atmosphere containing 5% CO_2_. Cells were subcultured when they reached approximately 80–90% confluence.

Drug-resistant derivatives were generated over a 6-month period by progressively increasing exposure to the corresponding EGFR-TKI. Specifically, the osimertinib concentration applied to parental H1975 cells was gradually raised from 10 nM to 2 μM, whereas gefitinib exposure in parental PC-9 cells was increased from 5 nM to 5 μM. To preserve the resistant phenotype, PC-9/GR and H1975/OR cells were maintained in medium containing 1 μM gefitinib (MedChemExpress, Monmouth Junction, NJ, USA; Cat. No. HY-50895) or 1 μM osimertinib (MedChemExpress; Cat. No. HY-15772), respectively. To minimize acute drug-related effects, resistant cells underwent a washout period of at least 48 h in EGFR-TKI-free medium before functional assays.

Cells were passaged at approximately 80–90% confluence and cryopreserved in 90% FBS and 10% dimethyl sulfoxide (DMSO; Solarbio, Beijing, China; Cat. No. D8371).

### 2.4 Genetic Modulation of cGAS, SIRT3, and Nrf2

cGAS expression was manipulated in opposite directions according to cellular drug sensitivity. Sensitive PC-9 and H1975 cells received lentiviral sh-cGAS or the corresponding negative-control short hairpin ribonucleic acid (shRNA, sh-NC), whereas resistant PC-9/GR and H1975/OR cells were infected with either a cGAS-overexpressing lentivirus or its matched empty-vector control. The cGAS knockdown and overexpression lentiviral vectors were constructed by Tsingke Biotechnology Co., Ltd. (Beijing, China). For cGAS knockdown, lentiviral transduction was performed directly at a multiplicity of infection (MOI) of 10 without polybrene. For cGAS overexpression, lentiviral transduction was performed at the same MOI in the presence of polybrene (8 μg/mL). At 8 h after lentiviral infection, the virus-containing medium was removed and replaced with fresh complete culture medium. Following an additional 48 h of culture, stable pooled cell populations were generated by selection with 2 μg/mL puromycin (Beyotime Biotechnology, Cat. No. ST551) for 7 days. The same puromycin selection procedure was applied to both cGAS knockdown and cGAS overexpression lentiviral transduction. Knockdown or overexpression efficiency was validated by Western blotting before subsequent experiments.

For SIRT3 modulation, sensitive PC-9 and H1975 cells were transfected with a SIRT3 overexpression plasmid or the corresponding empty vector control. The SIRT3 overexpression plasmid was constructed by GeneChem Co., Ltd. (Shanghai, China). Plasmid transfection was performed using Lipofectamine 3000 reagent (Invitrogen, Thermo Fisher Scientific, Waltham, MA, USA; Cat. No. L3000015) according to the manufacturer’s instructions. For cells seeded in 24-well plates, 0.8 μg/well pcDNA3.1-SIRT3 mammalian expression plasmid or the same amount of empty vector was used for transfection. Cells were harvested 48 h after plasmid transfection for subsequent analyses. The efficiency of SIRT3 overexpression was verified in parallel transfected cells by Western blotting before functional experiments.

SIRT3 expression in the resistant PC-9/GR and H1975/OR cells was suppressed using a small interfering RNA directed against SIRT3 (si-SIRT3), with a non-targeting siRNA serving as the negative control (si-NC). Both si-SIRT3 and si-NC were synthesized by GeneChem Co., Ltd. (Shanghai, China). Transfection was carried out with Lipofectamine RNAiMAX reagent (Invitrogen, Cat. No. 13778100) in accordance with the supplier’s protocol. si-SIRT3 and si-NC were each used at a final concentration of 30 nM, and cells were collected 72 h after transfection.

For activation of Nrf2 signaling, sensitive PC-9 and H1975 cells were transfected with either a pcDNA3.1-Nrf2 overexpression plasmid, a constitutively active Nrf2 construct, or the corresponding control vector. The pcDNA3.1-Nrf2 plasmid was supplied by GeneChem Co., Ltd. (Shanghai, China), whereas the constitutively active Nrf2 construct was generated by Hanbio Biotechnology Co., Ltd. (Shanghai, China). Lipofectamine 3000 reagent was used for plasmid delivery according to the manufacturer’s instructions. In 24-well plates, each well received 0.5 μg of pcDNA3.1-Nrf2 plasmid, 0.5 μg of constitutively active Nrf2 plasmid, or an equivalent quantity of the matched empty vector. Cells were harvested 48 h after transfection.

To inhibit Nrf2 expression, PC-9/GR and H1975/OR cells were transfected with Nrf2-specific siRNA (si-Nrf2) or the corresponding non-targeting control siRNA. GeneChem Co., Ltd. synthesized both oligonucleotides. Lipofectamine RNAiMAX was used for siRNA delivery following the manufacturer’s procedure. The final concentration of si-Nrf2 or si-NC was 30 nM, and experimental analyses were performed 72 h after transfection.

Rescue experiments were performed in cGAS-overexpressing resistant cells by co-modulating SIRT3 or Nrf2 as indicated. The shRNA and siRNA sequences are listed in Table 2. Knockdown and overexpression efficiencies were validated by Western blotting. Three independent biological replicates were included for every lentiviral, plasmid, and siRNA manipulation. Replicates were generated from separately seeded cultures that underwent transduction or transfection independently.

**Table 2:** RNA interference sequences used for cGAS, Nrf2, and SIRT3 knockdown in human NSCLC cells.

Target Protein	Gene Symbol	Knockdown Strategy	Sequence Information	Recommended Control
cGAS	CGAS/MB21D1	Lentiviral shRNA-mediated stable knockdown	sh-cGAS target sequence: 5′-CAACTACGACTAAAGCCATTT-3′	Non-targeting shRNA control (sh-NC)
Nrf2	NFE2L2/Nrf2	siRNA-mediated transient knockdown	si-Nrf2 sense: 5′-UCCCGUUUGUAGAUGACAA-3′; antisense: 5′-UUGUCAUCUACAAACGGGA-3′	Scrambled siRNA control (si-NC)
SIRT3	SIRT3	siRNA-mediated transient knockdown	si-SIRT3-1 sense: 5′-CCAGCAUGAAAUACAUUUATT-3′; antisense: 5′-UAAAUGUAUUUCAUGCUGGTT-3′	Scrambled siRNA control (si-NC)

Abbreviations: AMP, adenosine monophosphate; cGAS, cyclic GMP–AMP synthase; GMP, guanosine monophosphate; MB21D1, Mab-21 domain-containing protein 1; NC, negative control; NFE2L2/Nrf2, nuclear factor erythroid 2-related factor 2; NSCLC, non-small cell lung cancer; RNA, ribonucleic acid; shRNA, short hairpin ribonucleic acid; siRNA, small interfering ribonucleic acid; SIRT3, sirtuin 3.

### 2.5 Tumor Xenograft Model

The experimental design and reporting of the animal study followed the ARRIVE Essential 10 framework. Male BALB/c nude mice, 4–6 weeks of age and weighing 18–22 g, were purchased from Cavens Laboratory Animal Co., Ltd. (Changzhou, China). Animals were maintained under specific pathogen-free conditions at 22 ± 2°C and 50 ± 10% relative humidity, with a 12-h light/12-h dark cycle and unrestricted access to standard food and water. The experimental protocol was reviewed and approved by the Laboratory Animal Ethics Committee of Chongqing Medical University (IACUC-CQMU-2024-07083). Seventy-two mice were randomly distributed among 12 experimental groups, with six animals assigned to each group: H1975-control, H1975-sh-NC, H1975-sh-cGAS, H1975/OR-control, H1975/OR-OE-NC, H1975/OR-OE-cGAS, PC-9-control, PC-9-sh-NC, PC-9-sh-cGAS, PC-9/GR-control, PC-9/GR-OE-NC, and PC-9/GR-OE-cGAS.

For subcutaneous tumor establishment, cells at approximately 80% confluence were harvested and resuspended in an equal-volume mixture of phosphate-buffered saline and Matrigel (Corning, NY, USA; Cat. No. 356234). Each mouse received 5 × 10^6^ cells in a total injection volume of 100 μL into the flank. Tumor dimensions were recorded three times weekly with calipers by investigators blinded to the group assignments whenever practicable. Tumor volume was determined using the equation: volume = length × width^2^/2. Animals were euthanized upon reaching the predetermined humane endpoint or the maximum allowable tumor burden, or when severe distress, tumor ulceration, impaired mobility, or substantial body-weight loss was observed. At study termination, xenografts were removed, photographed, weighed, and prepared for subsequent histological and biochemical examinations.

### 2.6 Immunohistochemistry

Excised xenograft tissues were immersed in 4% paraformaldehyde (Solarbio, Cat. No. P1110), processed for paraffin embedding, and cut into 4-μm sections. After removal of paraffin and sequential rehydration, heat-mediated antigen retrieval was performed in citrate buffer at pH 6.0. Endogenous peroxidase activity was blocked by incubating the sections with hydrogen peroxide solution for 10 min at room temperature, followed by washing with PBS. Nonspecific binding sites were blocked with 5% bovine serum albumin (BSA; Beyotime Biotechnology, Cat. No. ST025) for 60 min at 37°C. The sections were then exposed overnight at 4°C to primary antibodies recognizing Ki-67, MMP9, GPX4, xCT, or ACSL4; antibody specifications are provided in Table 3.

**Table 3:** Antibodies used for immunohistochemistry.

Target	Antibody (Supplier, Catalog Cat. No.)	Dilution
Ki-67	Ki-67 Rabbit Polyclonal antibody (Proteintech, 27309-1-AP)	1:5000
MMP9	MMP-9 Rabbit Polyclonal antibody (Proteintech, 10375-2-AP)	1:200
ACSL4	ACSL4/FACL4 Rabbit Polyclonal antibody (Proteintech, 22401-1-AP)	1:200
GPX4	GPX4 Mouse Monoclonal antibody (Proteintech, 67763-1-Ig)	1:1000
xCT	SLC7A11/xCT Rabbit Polyclonal antibody (Proteintech, 26864-1-AP)	1:200

Abbreviations: ACSL4, acyl-CoA synthetase long chain family member 4; FACL4, fatty acid-CoA ligase 4; Cat. No., catalog number; CoA, coenzyme A; GPX4, glutathione peroxidase 4; Ki-67, proliferation marker protein Ki-67; MMP9, matrix metalloproteinase 9; SLC7A11, solute carrier family 7 member 11; xCT, cystine/glutamate antiporter encoded by SLC7A11.

After PBS washes, the slides were treated with reaction enhancer solution for 20 min at room temperature and subsequently incubated for another 20 min with an enhanced enzyme-conjugated goat anti-mouse/rabbit immunoglobulin G (IgG) polymer (ZSGB-BIO, Beijing, China; Cat. No. PV-9000). Immunoreactivity was visualized using 3,3′-diaminobenzidine substrate (DAB; Beyotime, Cat. No. P0202) for 5–8 min. Sections were then counterstained with hematoxylin, dehydrated, cleared, and coverslipped. Whole-slide images were obtained with an Olympus VS200 slide scanner (Olympus Corporation, Tokyo, Japan) under uniform acquisition settings. Semi-quantitative scoring included six tumors per condition; three sections from every tumor and five non-overlapping fields per section were sampled using random field selection. ImageJ software (version 1.53; National Institutes of Health, Bethesda, MD, USA) was used to quantify staining, which was reported as average optical density.

### 2.7 Western Blot Analysis

Cellular proteins were extracted on ice with radioimmunoprecipitation assay buffer (RIPA; Beyotime, Cat. No. P0013B) containing protease and phosphatase inhibitor mixtures (Beyotime, Cat. No. P1048). Total protein content was measured with a bicinchoninic acid assay kit (BCA; Beyotime, Cat. No. P0010S). For each lane, 30 μg of protein was resolved on an 8–12% sodium dodecyl sulfate–polyacrylamide gel and electrotransferred onto a polyvinylidene fluoride membrane (Millipore, Billerica, MA, USA; Cat. No. IPFL00010).

Following transfer, membranes were blocked for 1 h at room temperature. Membranes intended for the detection of phosphorylated proteins, including p-Nrf2 (Ser40), p-EGFR, p-AKT1, p-ERK1/2, and γ-H2AX, were blocked only with 5% BSA prepared in Tris-buffered saline containing 0.1% Tween-20. For non-phosphorylated targets, either 5% nonfat milk or 5% BSA in Tris-buffered saline containing 0.1% Tween 20 (TBST) was selected according to the requirements of the corresponding primary antibody. Primary antibodies against cGAS, SIRT3, Nrf2, p-Nrf2 (Ser40), EGFR, p-EGFR, AKT1, p-AKT1, ERK1/2, p-ERK1/2, γ-H2AX, BRCA1, RAD51, Ku70, Ku80, GPX4, xCT, ACSL4, β-actin, and Histone H3 were used as detailed in Table 4.

**Table 4:** Antibodies used for western blotting.

Target	Antibody (Supplier, Catalog Cat. No.)	Dilution
cGAS	Rabbit anti-cGAS (Proteintech, 26416-1-AP)	1:1000
BRCA1	Rabbit anti-BRCA1 (Proteintech, 22362-1-AP)	1:1000
RAD51	Rabbit anti-RAD51 (Proteintech, 14961-1-AP)	1:2000
Ku70	Rabbit anti-Ku70 (Proteintech, 10723-1-AP)	1:1000
Ku80	Rabbit anti-Ku80 (Proteintech, 16389-1-AP)	1:1000
GPX4	GPX4 Mouse Monoclonal antibody (Proteintech, 67763-1-Ig)	1:1000
xCT	SLC7A11/xCT Rabbit Polyclonal antibody (Proteintech, 26864-1-AP)	1:1000
ACSL4	ACSL4/FACL4 Rabbit Polyclonal antibody (Proteintech, 22401-1-AP)	1:1000
SIRT3	Rabbit anti-SIRT3 (Proteintech, 10099-1-AP)	1:2000
Nrf2	Rabbit anti-Nrf2 (Proteintech, 16396-1-AP)	1:2000
p-Nrf2 (Ser40)	Rabbit anti-p-Nrf2 (HUABIO, Hangzhou, China; ET1608-28)	1:1000
β-actin	Mouse anti-β-actin (Proteintech, 66009-1-Ig)	1:5000
γ-H2AX	Rabbit anti-gamma H2AX (Abcam, ab81299)	1:1000
Histone H3	Histone H3 Polyclonal antibody (Proteintech, 17168-1-AP)	1:5000
EGFR	EGFR Recombinant Rabbit Monoclonal Antibody (HUABIO, ET1604-44)	1:1000
p-EGFR	Phospho-EGFR Recombinant Rabbit Monoclonal Antibody (HUABIO, ET1611-50)	1:1000
AKT1	AKT1 Recombinant Rabbit Monoclonal Antibody (HUABIO, ET1609-47)	1:1000
p-AKT1	Phospho-AKT1 Recombinant Rabbit Monoclonal Antibody (HUABIO, ET1701-36)	1:1000
ERK1/2	ERK1/2 Recombinant Rabbit Monoclonal Antibody (HUABIO, ET1601-29)	1:1000
p-ERK1/2	Phospho-Erk1 (T202 + Y204) + Erk2 (T185 + Y187) Recombinant Rabbit Monoclonal Antibody (HUABIO, ET1610-13)	1:1000

Abbreviations: ACSL4, acyl-CoA synthetase long-chain family member 4; AKT1, AKT serine/threonine kinase 1; AMP, adenosine monophosphate; BRCA1, BRCA1 DNA repair-associated protein; Cat. No., catalog number; cGAS, cyclic GMP–AMP synthase; CoA, coenzyme A; EGFR, epidermal growth factor receptor; ERK1/2, extracellular signal-regulated kinases 1 and 2; FACL4, long-chain fatty acid-CoA ligase 4; GMP, guanosine monophosphate; GPX4, glutathione peroxidase 4; γ-H2AX, histone H2AX phosphorylated at serine 139; Ku70, Ku autoantigen 70-kDa subunit; Ku80, Ku autoantigen 80-kDa subunit; Nrf2, nuclear factor erythroid 2-related factor 2; p-, phosphorylated; RAD51, RAD51 recombinase; Ser, serine; SIRT3, sirtuin 3; SLC7A11, solute carrier family 7 member 11; T, threonine; xCT, cystine/glutamate antiporter encoded by SLC7A11; Y, tyrosine. β-actin, beta-actin.

After incubation with the primary antibodies and subsequent washing, membranes were exposed for 1 h at room temperature to horseradish peroxidase-linked secondary antibodies. These included horseradish peroxidase (HRP)-conjugated goat anti-rabbit IgG (Abbkine, Wuhan, China; Cat. No. A21020; 1:5000) and HRP-conjugated goat anti-mouse IgG (Abbkine; Cat. No. A21010; 1:5000). Chemiluminescent signals were generated with an enhanced chemiluminescence reagent (4A Biotech, Beijing, China; Cat. No. 4AW011) and recorded using a SOLO 6S chemiluminescence imaging system (VILBER, Paris, France) under exposure conditions that avoided signal saturation. Band density was measured in ImageJ version 1.53. Target protein abundance was normalized to β-actin, Histone H3, or the corresponding total protein, as applicable. Protein measurements were reproduced in three or more separately prepared cell cultures.

### 2.8 Nuclear and Cytoplasmic Protein Fractionation

Cytoplasmic and nuclear proteins were separated with a Nuclear and Cytoplasmic Protein Extraction Kit (Beyotime, Cat. No. P0028) according to the protocol supplied with the kit. Following the indicated experimental treatments, cells were harvested and rinsed twice with ice-cold PBS. The resulting cell pellets were suspended in cytoplasmic extraction reagent containing protease and phosphatase inhibitors and maintained on ice. After extraction of the cytoplasmic components, the samples were centrifuged at 12,000× *g* for 5 min at 4°C. The supernatant was removed carefully and retained as the cytoplasmic fraction, while avoiding disruption of the nuclear pellet.

The residual pellet was washed and subsequently resuspended in nuclear extraction reagent supplemented with protease and phosphatase inhibitors. After repeated vortexing and incubation on ice, the samples were centrifuged at 12,000× *g* for 10 min at 4°C, and the resulting supernatant was collected as the nuclear fraction. Protein concentrations in both fractions were determined by bicinchoninic acid assay. Equal quantities of cytoplasmic or nuclear protein were analyzed by Western blotting for Nrf2 and p-Nrf2 (Ser40). β-actin served as the cytoplasmic loading control, whereas Histone H3 was used to verify and normalize the nuclear fraction.

### 2.9 Quantitative Real-Time PCR

Cellular RNA was isolated with TRIzol reagent (Invitrogen, Thermo Fisher Scientific; Cat. No. 15596026) following the procedure recommended by the manufacturer. RNA quantity and purity were determined by spectrophotometric measurement. Before complementary DNA (cDNA) synthesis, the extracted RNA was incubated with ribonuclease (RNase)-free deoxyribonuclease I (DNase I) to remove residual genomic DNA. For each specimen, 1 μg of total RNA was converted into cDNA in a 20-μL reaction using PrimeScript RT Master Mix (Takara; Cat. No. RR036A). The reverse-transcription program consisted of incubation at 37°C for 15 min and enzyme inactivation at 85°C for 5 s.

Quantitative PCR amplification was carried out with TB Green Premix Ex Taq II (Takara; Cat. No. RR820A) on a QuantStudio 5 Real-Time PCR System (Applied Biosystems, Thermo Fisher Scientific, Waltham, MA, USA). Each reaction had a final volume of 20 μL and contained 10 μL of 2× TB Green Premix Ex Taq II, 0.8 μL each of forward and reverse primers, 0.4 μL of 6-carboxy-X-rhodamine (ROX) Reference Dye II, 2 μL of cDNA, and 6 μL of nuclease-free water. Amplification began with denaturation at 95°C for 30 s, followed by 40 cycles consisting of 95°C for 5 s and 60°C for 30 s. A melting-curve analysis was performed after amplification to confirm the specificity of the products. Transcript abundance was determined by the 2^−ΔΔCt^ method, using β-actin for normalization.

The oligonucleotide sequences used for amplification were: cGAS forward, 5′-CAGCCACAGAGGAAGACAGC-3′, and reverse, 5′-GGCATCTTCTCCACCTTCCT-3′; SIRT3 forward, 5′-GCTGGCCTCAGATGGTGTCTG-3′, and reverse, 5′-CGGTGGTGGTGAAGATGACA-3′; and β-actin forward, 5′-CATGTACGTTGCTATCCAGGC-3′, and reverse, 5′-CTCCTTAATGTCACGCACGAT-3′. Measurements for each biological specimen were obtained in technical triplicate, and a minimum of three independently conducted experiments were analyzed.

### 2.10 Co-Immunoprecipitation Analysis

Co-immunoprecipitation was performed to examine whether the indicated proteins were present in the same protein complex. cGAS-overexpressing H1975/OR cells and their matched control cells were rinsed twice with ice-cold PBS and subsequently disrupted in a non-denaturing immunoprecipitation buffer containing protease and phosphatase inhibitors. The lysates were maintained on ice for 30 min and then centrifuged at 12,000× *g* for 15 min at 4°C to remove insoluble material. The protein concentration of each clarified lysate was quantified using a BCA assay.

For each pull-down reaction, 500 μg of total cellular protein was brought to the same volume with lysis buffer. To reduce nonspecific binding, the lysates were first incubated with 30 μL of protein A/G magnetic beads (MedChemExpress; Cat. No. HY-K0202) for 1 h at 4°C under gentle rotation. After removal of the beads by magnetic separation, the precleared lysates were incubated overnight at 4°C with 2 μg of the designated antibody per 500 μg protein. The antibodies used were anti-cGAS (Proteintech, Wuhan, China; Cat. No. 26416-1-AP), anti-SIRT3 (Proteintech; Cat. No. 10099-1-AP), and anti-Nrf2 (Proteintech, Cat. No. 16396-1-AP). Parallel negative-control reactions received 2 μg of normal IgG (Cell Signaling Technology, Danvers, MA, USA; Cat. No. 3900 or 5415) under identical conditions. Thereafter, 30 μL of fresh protein A/G magnetic beads was introduced into each reaction, and incubation was continued for 2 h at 4°C with rotation to recover the immune complexes.

The magnetic beads were retrieved on a magnetic rack and rinsed five times with chilled lysis buffer. Proteins retained on the beads were released in sodium dodecyl sulfate (SDS) sample buffer by heating at 95°C for 5 min. The immunoprecipitated fractions, together with 10% of the corresponding input lysates, were subjected to Western blot analysis using the specified antibodies. Each co-immunoprecipitation experiment was repeated independently at least three times.

### 2.11 SIRT3 Activity Assay

SIRT3 deacetylase activity was quantified using a fluorometric SIRT3 Activity Assay Kit (Abcam, Cambridge, UK; Cat. No. ab156067) following the manufacturer’s recommended protocol. Briefly, PC-9 and H1975 cells were subjected to cGAS knockdown, Nrf2 overexpression, or SIRT3 overexpression, whereas PC-9/GR and H1975/OR cells were subjected to cGAS overexpression, Nrf2 knockdown, or SIRT3 knockdown, as indicated. Corresponding negative controls, empty vectors, and vehicle controls were included.

Mitochondrial fractions were freshly isolated immediately before measurement of SIRT3 enzymatic activity. Cells were rinsed twice with chilled PBS, harvested by centrifugation, and suspended in ice-cold mitochondrial isolation buffer. The suspensions were homogenized on ice, after which the homogenates were centrifuged at 600× *g* for 10 min at 4°C to sediment nuclei and intact cells. The collected supernatants were subjected to a second centrifugation at 10,000× *g* for 15 min at 4°C to obtain the mitochondrial pellets. These pellets were washed once with mitochondrial isolation buffer and centrifuged again at 10,000× *g* for 10 min at 4°C. The purified mitochondrial material was then lysed in the buffer supplied for the SIRT3 assay, and mitochondrial protein content was quantified by BCA analysis. To limit protein degradation and contamination by cytosolic components, the entire isolation procedure was conducted on ice.

For each reaction, 10 μg of mitochondrial protein was loaded per well in a black 96-well microplate. Each 50 μL reaction contained mitochondrial protein sample, SIRT3 assay buffer, fluoro-substrate peptide, NAD, developer, and nuclease-free water according to the manufacturer’s reaction system. Recombinant SIRT3 provided in the kit was used as a positive control, whereas no-enzyme, no-sample, and no-NAD controls were included for background correction. Reactions were initiated by adding mitochondrial protein samples and were mixed thoroughly at room temperature.

Fluorescence was recorded for 60 min at 2 min intervals with a Varioskan LUX multimode microplate reader (Thermo Fisher Scientific, Waltham, MA, USA). Excitation and emission wavelengths were set to 350 and 450 nm, respectively. Enzymatic activity was derived from the initial linear segment of the fluorescence-time curve. The signal obtained from the no-enzyme control was subtracted as background, and the resulting SIRT3 activity was normalized to the amount of mitochondrial protein analyzed.

### 2.12 Cell Counting Kit-8 Assay

The effects of EGFR-TKIs on cell viability were determined with a Cell Counting Kit-8 assay (CCK-8; Dojindo, Kumamoto, Japan; Cat. No. CK04). Cells were distributed into 96-well plates at 5 × 10^3^ cells per well in 100 μL of complete medium and maintained overnight to permit attachment. The following day, the cultures were exposed for 24 h to serial concentrations of gefitinib or osimertinib. Gefitinib was tested at 1, 5, 10, 25, 50, and 100 μM, whereas osimertinib was applied at 1, 2.5, 5, 10, 25, and 50 μM.

At the end of drug exposure, 10 μL of CCK-8 reagent was dispensed into each well, and the plates were returned to 37°C for 2 h. Optical density was subsequently recorded at 450 nm, using 650 nm as the reference wavelength, on a Varioskan LUX multimode microplate reader (Thermo Fisher Scientific, Waltham, MA, USA). Viability was expressed relative to that of the untreated control cells. Dose–response relationships were fitted by nonlinear regression in GraphPad Prism version 10.0 (GraphPad Software, Boston, MA, USA), from which half-maximal inhibitory concentration values were obtained. Every treatment condition was assessed in three technical wells, and the complete experiment was independently repeated at least three times.

### 2.13 EdU Proliferation Assay

DNA synthesis was examined using an EdU incorporation kit (Beyotime, Cat. No. C0078S). Cells were dispensed into 24-well plates at 5 × 10^4^ cells per well and allowed to attach overnight. Following the designated 24-h treatments, 10 μM EdU was introduced during the final 2 h of incubation.

The cultures were subsequently immersed in 4% paraformaldehyde (Solarbio, Cat. No. P1110) for 15 min at room temperature. After PBS rinsing, cellular membranes were permeabilized with 0.5% Triton X-100 for 15 min. The click-reaction solution supplied with the kit was applied for 30 min at room temperature under light-protected conditions. Nuclear DNA was visualized by a 5-min incubation with DAPI (Beyotime Biotechnology, Cat. No. C1002; final concentration, 1 μg/mL). A Leica DM IL LED microscope was used to record EdU and nuclear signals, with exposure, gain, and magnification held constant for all comparison groups. Three wells were evaluated for each condition, and the complete procedure was reproduced in at least three independent experiments.

### 2.14 Immunofluorescence Staining

Sterile coverslips placed in 24-well plates were used for cell culture and the indicated experimental interventions. At the end of treatment, the cells were rinsed with PBS, preserved in 4% paraformaldehyde for 15 min, and exposed to 0.3% Triton X-100 for 10 min at room temperature. A 1-h incubation with 10% normal goat serum (Beyotime, Cat. No. C0265) was used to reduce nonspecific antibody binding.

The specimens were then maintained at 4°C overnight with antibodies against γ-H2AX (Abcam, Cat. No. ab81299; 1:200), cGAS (Proteintech, Cat. No. 26416-1-AP; 1:200), SIRT3 (Proteintech, Cat. No. 10099-1-AP; 1:200), or Nrf2 (Proteintech, Cat. No. 16396-1-AP; 1:200). Following PBS washing, the coverslips were incubated with a DyLight 594-conjugated goat anti-rabbit IgG secondary antibody (Abbkine, Cat. No. A23420; 1:500) for 1 h at room temperature in the dark. DAPI (Beyotime, Cat. No. C1002) was subsequently applied at 1 μg/mL for 5 min, after which the coverslips were mounted in antifade medium.

Fields were captured on the Leica DM IL LED platform using a prespecified acquisition configuration that was not altered between experimental conditions. Signal intensity was determined in ImageJ version 1.53 (National Institutes of Health). No fewer than five randomly chosen fields were included for each experimental group.

### 2.15 Wound-Healing Assay

Lateral cell motility was assessed by monitoring closure of a mechanically generated gap. Cells were plated in 6-well dishes at 5 × 10^5^ cells per well and cultured to approximately 90% confluence. A straight wound was created through the monolayer with a sterile 200 μL pipette tip. Floating cells were eliminated by two gentle PBS washes.

To limit the contribution of proliferation to gap closure, the wounded monolayers were maintained in serum-free basal medium. PC-9 and PC-9/GR cultures received serum-free DMEM (Gibco, Thermo Fisher Scientific, Grand Island, NY, USA; Cat. No. 31870074), whereas serum-free RPMI-1640 medium (Gibco, Thermo Fisher Scientific; Cat. No. 31870082) was used for H1975 and H1975/OR cells. The wounded areas were imaged immediately and again at 12 and 24 h with a Leica DM IL LED inverted laboratory microscope. ImageJ version 1.53 was used to measure residual wound area or width, and closure was calculated relative to the corresponding value at 0 h. Each treatment was tested in three wells, with at least three independent repetitions.

### 2.16 Transwell Migration and Invasion Assays

Motility through a porous membrane was measured in 24-well Transwell units fitted with 8-μm polycarbonate filters (Corning, Cat. No. 3422). For migration analysis, 1 × 10^5^ cells in serum-free basal medium were introduced into the upper compartment. The lower compartment was filled with 600 μL complete medium supplemented with 20% FBS. After 24 h at 37°C, nonmigrated cells were removed from the upper membrane surface.

For the invasion experiment, each insert was first coated with 50 μL Matrigel basement membrane matrix (Corning, Cat. No. 356234) diluted 1:8 in chilled serum-free basal medium. Polymerization was allowed to proceed for 1 h at 37°C. Thereafter, 4 × 10^5^ cells in serum-free basal medium were loaded above the matrix, while 600 μL complete medium containing 20% FBS (PAN-Biotech, Cat. No. P30-3302) was placed below. The invasion interval was 24 h.

Cells that had reached the underside of the membrane were preserved in 4% paraformaldehyde for 15 min and colored with 0.1% crystal violet for 20 min at room temperature. Representative fields were photographed on a Leica DM IL LED inverted microscope. Five randomly selected areas from each insert were counted in ImageJ version 1.53. Technical triplicates were included, and each assay was conducted independently at least three times.

### 2.17 Transmission Electron Microscopy

For ultrastructural examination, cells were initially preserved for 2 h at 4°C in 2.5% glutaraldehyde prepared in 0.1 M sodium cacodylate buffer. Secondary fixation was performed with 1% osmium tetroxide for 1 h. The specimens were dehydrated sequentially through increasing concentrations of ethanol and acetone before infiltration and embedding in epoxy resin.

Sections approximately 70 nm thick were cut from the resin blocks and contrasted with uranyl acetate followed by lead citrate. Cellular ultrastructure was examined at 80 kV using a JEM-1400Plus transmission electron microscope (JEOL Ltd., Tokyo, Japan). Digital images were recorded with the integrated 8-megapixel charge-coupled device (CCD) system. Evaluation of the micrographs was undertaken without knowledge of the sample allocation.

### 2.18 Nuclear and Mitochondrial DNA Assays

Cells were seeded into glass-bottom dishes at 1 × 10^5^ cells per well. Before fixation, mitochondria were labeled for 30 min at 37°C with 200 nM MitoTracker Red CMXROS (Thermo Fisher Scientific, Cat. No. M7512) diluted in serum-free DMEM (Gibco, Cat. No. 31870074). The cells were then fixed in 4% paraformaldehyde for 15 min, permeabilized in 0.5% Triton X-100 for 10 min, and blocked for 1 h with 5% BSA (Beyotime Biotechnology, Cat. No. ST025).

Double-stranded DNA was detected by incubation with an anti-dsDNA antibody (Santa Cruz Biotechnology, Dallas, TX, USA; Cat. No. sc-58749; 1:400) for 1 h at 37°C. Alexa Fluor 488-conjugated goat anti-mouse IgG (Abbkine, Cat. No. A23210; 1:500) was applied for a further 1 h at room temperature in the dark. Nuclear counterstaining was performed for 5 min using ready-to-use DAPI solution (10 μg/mL; Solarbio, Cat. No. C0065). A Leica DM IL LED inverted laboratory microscope was used for image acquisition with unchanged exposure parameters. The optical settings were 405-nm excitation and 410–480-nm emission for DAPI, 488-nm excitation and 500–550-nm emission for Alexa Fluor 488, and 561-nm excitation and 570–620-nm emission for MitoTracker Red. At least three independently prepared experiments were examined.

### 2.19 Quantification of Double-Stranded DNA

The amount of intracellular double-stranded DNA (dsDNA) was determined with PicoGreen dsDNA quantitation reagent (Yeasen Biotechnology, Shanghai, China; Cat. No. 12641ES01). Cells were rinsed twice in ice-cold PBS and disrupted in a buffer composed of 10 mM Tris-HCl, 10 mM ethylenediaminetetraacetic acid (EDTA), and 0.5% SDS. To dissociate DNA from associated proteins, proteinase K was added to 100 μg/mL, and the lysates were maintained at 55°C for 1 h.

Following centrifugation at 12,000× *g* for 10 min, the DNA-containing supernatants were recovered. Samples and DNA standards were combined with the PicoGreen working reagent in black 96-well plates, with a final reaction volume of 200 μL. After 2 min at room temperature in the dark, fluorescence was read at 480-nm excitation and 530-nm emission on a Varioskan LUX multimode microplate reader (Thermo Fisher Scientific). Concentrations were derived from the standard curve and adjusted to the relevant protein content or cell number. The analysis was reproduced in at least three separate experiments.

### 2.20 Ferrous Ion Detection

The labile intracellular Fe^2^^+^ pool was visualized with FerroOrange (Dojindo, Cat. No. F374). Cells were plated in 6-well dishes at 2 × 10^5^ cells per well and allowed to reach approximately 70–80% confluence. After completion of the assigned treatments, serum-free medium was used to rinse the cells, followed by incubation with 1 μM FerroOrange for 30 min at 37°C in darkness.

Because post-staining washing was omitted, images were obtained immediately after incubation. A Leica DM IL LED inverted laboratory microscope was operated with fixed exposure settings, and fluorescence was collected through the red channel at approximately 543-nm excitation and 580-nm emission.

### 2.21 Intracellular and Mitochondrial ROS Detection

CellROX Orange (Thermo Fisher Scientific, Cat. No. C10443) and MitoSOX Red (Thermo Fisher Scientific, Cat. No. M36008) were used to monitor total intracellular and mitochondrial reactive oxygen species, respectively. Following the indicated treatments, cells designated for mitochondrial superoxide detection were washed with PBS and incubated with 5 μM MitoSOX Red in serum-free, phenol red-free medium for 30 min at 37°C in the dark. In parallel, independently cultured cells designated for intracellular ROS detection were washed with PBS and incubated with 5 μM CellROX Orange in the same type of medium for 30 min under identical temperature and light-protected conditions. After probe incubation, the cells were gently washed with PBS and counterstained with Hoechst 33342 for 5 min at 37°C. Excess dye was removed by washing with PBS.

Fluorescence images were acquired separately for CellROX Orange- and MitoSOX Red-stained cells using a Leica DM IL LED inverted fluorescence microscope. Probe-specific acquisition settings were used for the two dyes, whereas the exposure time, gain, illumination intensity, and other imaging parameters were kept constant across the experimental groups stained with the same probe. Three independently prepared cell cultures were examined for each experimental condition.

### 2.22 Mitochondrial Membrane Potential (ΔΨm) Assay

Changes in mitochondrial membrane potential were examined using the JC-1 kit (Beyotime, Cat. No. C2003S). After the indicated interventions, PC-9/GR, H1975/OR, and cGAS- or SIRT3-modified cells were washed with PBS. JC-1 working solution was prepared at 2 μM in serum-free DMEM (Gibco, Cat. No. 31870074) and applied for 30 min at 37°C in the dark.

The cultures were then rinsed twice with the kit-provided JC-1 buffer and immediately examined with a Leica DM IL LED inverted laboratory microscope. Aggregated JC-1 was recorded at approximately 514-nm excitation and 590-nm emission, whereas the monomeric signal was obtained at approximately 488/530 nm. The aggregate and monomer channels were recorded without altering the acquisition settings between groups, and the assay was performed in three independently treated cultures.

### 2.23 Lipid Peroxide Detection

Membrane lipid oxidation was assessed using C11 BODIPY 581/591 (GlpBio, Montclair, CA, USA; Cat. No. GC40165). PC-9, H1975, the corresponding resistant derivatives, and cells subjected to cGAS or SIRT3 modulation were exposed to 2 μM probe in serum-free, phenol red-free medium for 30 min at 37°C with protection from light.

Following PBS washing, nuclei were stained with Hoechst 33342 (Beyotime, Cat. No. C1027; 2 μg/mL) for 10 min. Images were acquired on a Leica DM IL LED inverted laboratory microscope using constant exposure conditions. The oxidized probe was detected through the green channel at approximately 488/520 nm, whereas the reduced form was recorded through the red channel at approximately 543/590 nm. Oxidation measurements were obtained from at least three separately prepared biological samples.

### 2.24 Ferrostatin-1 Rescue Assay

The contribution of ferroptotic injury to the observed redox phenotype was further tested using Ferrostatin-1 (Fer-1; MedChemExpress, Cat. No. HY-100579). A final concentration of 1 μM Fer-1 was used throughout the rescue experiments. For EGFR-TKI-sensitive cells, Fer-1 and the corresponding tyrosine kinase inhibitor were administered concurrently. In resistant models, cGAS-overexpressing cells received Fer-1 either alone or together with the matched EGFR-TKI.

The intervention lasted 24 h. Thereafter, intracellular Fe^2^^+^, lipid oxidation, intracellular ROS, reduced glutathione, and malondialdehyde were assessed using the respective procedures described in the corresponding subsections. Microscopic fluorescence endpoints were recorded with the Leica DM IL LED platform, whereas plate-based biochemical signals were obtained with the Varioskan LUX multimode microplate reader, as applicable. Vehicle-treated cultures were included as the reference condition. Each rescue experiment was independently carried out at least three times.

### 2.25 Malondialdehyde Assay

Malondialdehyde was measured with a thiobarbituric acid-based kit (Beyotime, Cat. No. S0131S). Cultured cells were rinsed in chilled PBS and lysed on ice. For xenografts, freshly removed tumors were weighed, cut into small pieces, and homogenized in cold lysis buffer. All tissue processing was performed on ice to limit artificial lipid oxidation.

After centrifugation at 12,000× *g* for 10 min at 4°C, the soluble fractions were retained. Protein concentration was measured with the BCA kit (Beyotime Biotechnology, Cat. No. P0010), and equivalent protein input was used for subsequent reactions. Samples or standards were combined with thiobarbituric acid reagent and heated at 95°C for 30 min. Once cooled to room temperature, the reaction mixtures were centrifuged at 1000× *g* for 10 min to remove particulate material.

The clarified solutions were transferred into 96-well plates. Absorbance at 532 nm was read using a Varioskan LUX multimode microplate reader (Thermo Fisher Scientific, Waltham, MA, USA). A second reading at 600 nm was obtained to correct for sample turbidity, and the net signal was calculated as A532–A600. Quantification was based on a 1,1,3,3-tetraethoxypropane calibration curve and expressed relative to total protein. Cell experiments included at least three independent replicates; where tumor tissue was examined, six xenografts from each group were included.

### 2.26 Glutathione Assay

Reduced glutathione was analyzed with the Solarbio assay kit (Cat. No. BC1175). Cell preparations were washed with cold PBS, enumerated, and extracted in the buffer supplied with the kit. Fresh xenograft samples were weighed, minced, and homogenized in the same ice-cold extraction solution.

Cell and tissue preparations were subjected to brief pulse sonication on ice and subsequently cleared at 12,000× *g* for 10 min at 4°C. To eliminate interference from protein-bound sulfhydryl groups, the resulting supernatants were deproteinized with the acidic precipitation reagent included in the kit. The clarified extracts were combined with the colorimetric reagents and maintained at room temperature for the interval specified by the manufacturer.

Optical density at 412 nm was measured on a Varioskan LUX multimode microplate reader. Reduced glutathione concentrations were obtained from serially diluted GSH standards. Tumor values were normalized to protein measured with a BCA assay (Beyotime Biotechnology, Cat. No. P0010) and expressed per milligram of protein. Values from cultured cells were adjusted to the corresponding cell count and reported per defined number of cells. Technical triplicates were analyzed for each specimen, and no fewer than three independent experiments were performed.

### 2.27 Alkaline Comet Assay

Single-cell DNA strand damage was evaluated under alkaline conditions with the OxiSelect Comet Assay Kit (Cell Biolabs, San Diego, CA, USA; Cat. No. STA-350). PC-9, H1975, PC-9/GR, and H1975/OR cells were collected, washed in chilled PBS, and resuspended at 1 × 10^5^ cells/mL. The suspension was combined with low-melting-point comet agarose at a ratio of 1:10. A 75 μL aliquot was immediately distributed into each well of a precoated comet slide.

The slides remained horizontal at 4°C in darkness for 30 min to allow gel formation. They were then transferred to prechilled lysis solution for 60 min at 4°C, followed by 30 min in cold alkaline solution to permit DNA unwinding. Electrophoresis was carried out in chilled alkaline running buffer for 20 min at 1 V/cm and 300 mA.

After electrophoresis, the slides were rinsed with distilled water, treated with 70% ethanol for 5 min, and dried for 30 min at 37°C. The 10,000× Vista Green DNA dye was diluted 1:10,000 in Tris-ethylenediaminetetraacetic acid (TE) buffer to prepare a 1× staining solution. After the agarose and slides had completely dried, 100 μL of the 1× Vista Green DNA staining solution was added to each well, followed by incubation at room temperature for 15 min in the dark. Comets were visualized using a Leica DM IL LED inverted laboratory microscope fitted with a FITC-compatible filter. Tail moment, tail DNA percentage, and olive tail moment were calculated in CometScore 2.0 software (TriTek Corporation, version 2.0, Sumerduck, VA, USA). A minimum of 50 cells was scored for each specimen, and three independent experiments were completed.

### 2.28 Statistical Analysis

GraphPad Prism 10.0 (GraphPad Software, Boston, MA, USA) was used for statistical testing and graphical presentation, whereas computational bioinformatics procedures were conducted in R version 4.5.1 (R Foundation for Statistical Computing). Unless specified otherwise, numerical results are reported as the mean ± standard deviation and represent at least three biologically independent experiments.

An unpaired, two-sided Student’s *t* test was applied when two independent groups were compared. Datasets comprising three or more groups were evaluated by one-way analysis of variance. Two-way analysis of variance was selected when the design contained two experimental factors, including drug concentration–response experiments. Half-maximal inhibitory concentrations were estimated by nonlinear regression. For gene set enrichment analysis, statistical significance was judged according to the FDR q value. A two-sided *p* value below 0.05 was regarded as significant. Significance symbols were assigned as follows: *p < 0.05, **p < 0.01, and ***p < 0.001.

## 3 Results

### 3.1 EGFR-TKI Treatment Is Associated with DNA Damage and cGAS Activation in NSCLC Cells

**Figure 1:**
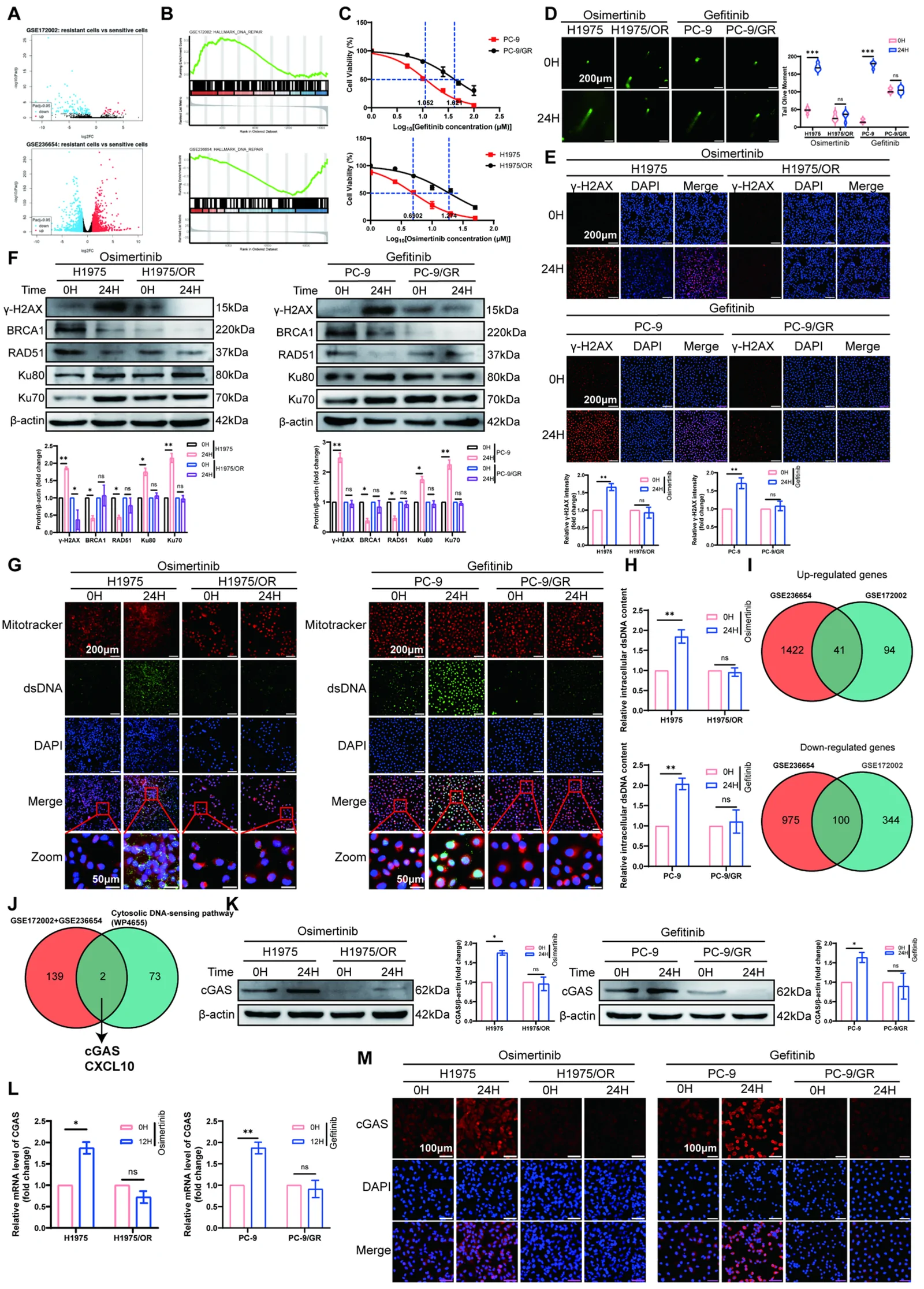
EGFR-TKI treatment is associated with DNA damage and cGAS activation in NSCLC cells. (**A**) Volcano maps of GSE172002 and GSE236654. (**B**) HALLMARK_DNA_REPAIR in GSEA enrichment analysis of GSE172002 and GSE236654. (**C**) IC_50_ values for PC-9, PC-9/GR, H1975, and H1975/OR cells. (**D**) Comet assay (tail olive moment) showing DNA damage in PC-9 and PC-9/GR cells after gefitinib, and in H1975 and H1975/OR cells after osimertinib (scale bar = 200 μm). (**E**) Immunofluorescence of γ-H2AX foci in the same treatment groups (scale bar = 200 μm). (**F**) Western blot of γ-H2AX, BRCA1, RAD51, Ku70, and Ku80 expression following EGFR-TKI treatment. (**G**) Representative MitoTracker, dsDNA, and DAPI staining showing cytosolic dsDNA distribution in PC-9, PC-9/GR, H1975, and H1975/OR cells (scale bars = 200 μm, 50 μm). (**H**) Quantification of cytoplasmic dsDNA after treatment. (**I**) Venn diagrams showing the overlap of upregulated and downregulated genes between GSE172002 and GSE236654, including 41 shared upregulated genes and 100 shared downregulated genes. (**J**) Venn diagram of the differentially expressed genes shared by GSE172002, GSE236654 and the WikiPathway Cytosolic DNA-sensing pathway (WP4655) genes. (**K**) Western blotting (WB) and (**L**) quantitative reverse transcription polymerase chain reaction (qRT-PCR) analysis of cGAS expression. (**M**) Immunofluorescence localization of cGAS (scale bar = 100 μm). Quantitative data were normalized to the corresponding 0H group within each cell line and are presented as fold change relative to the respective 0H control. For Western blot analysis, target protein levels were first normalized to β-actin before fold-change normalization. Error bars represent mean ± SD from at least three independent biological replicates. *p < 0.05, **p < 0.01, ***p < 0.001, *ns*, not significant.

Transcriptomic data from EGFR-TKI–resistant (osimertinib/gefitinib) cell lines in GEO were analyzed. Differentially expressed genes (DEGs) were defined by *p* value < 0.05 and |log2FC| ≥ 1. Volcano plots depict DEG distributions for GSE172002 and GSE236654 (Fig. 1A). Despite opposite enrichment directions, HALLMARK_DNA_REPAIR reached statistical significance in both cohorts, directing subsequent experiments toward treatment-associated genomic stress (GSE172002: NES = 1.407, FDR q = 0.00864; GSE236654: NES = −1.728, FDR q = 0.0000680) (Fig. 1B, Table 1).

We next validated the resistant phenotype in NSCLC cell models. Compared with parental PC-9 and H1975 cells, PC-9/GR and H1975/OR cells displayed reduced sensitivity to gefitinib and osimertinib, respectively, as shown by right-shifted dose–response curves and increased IC_50_ values (Fig. 1C). In parallel, EGFR-TKI treatment markedly reduced p-EGFR, p-AKT1, and p-ERK1/2 levels in parental cells, whereas these inhibitory effects were less evident in the corresponding resistant cells (Supplementary Fig. S1A,B). Collectively, the rightward displacement of the viability curves and persistence of downstream phosphorylation verified the resistant phenotype of both derivative lines.

Based on the GSEA results, we further examined DNA damage-related changes after EGFR-TKI exposure. Comet assay showed increased DNA tail formation in H1975 cells treated with osimertinib and in PC-9 cells treated with gefitinib, while this change was relatively weaker in H1975/OR and PC-9/GR cells (Fig. 1D). Consistently, γ-H2AX immunofluorescence showed stronger DNA damage signals in parental cells after EGFR-TKI treatment than in resistant cells (Fig. 1E). Western blot analysis further showed that EGFR-TKI exposure increased γ-H2AX expression and altered the expression of DNA repair-related proteins, including BRCA1, RAD51, Ku70, and Ku80, mainly in parental cells, whereas these treatment-induced changes were less evident in resistant cells (Fig. 1F). Thus, matched TKI exposure generated a stronger genomic-stress response in the parental populations than in their resistant derivatives.

Since DNA damage may lead to cytosolic DNA accumulation and cGAS activation, we next evaluated cytosolic dsDNA and cGAS expression. Fluorescence staining showed increased cytosolic dsDNA signals after osimertinib or gefitinib treatment in H1975 and PC-9 cells, respectively. Consistently, dsDNA quantification showed a greater increase in parental cells than in resistant cells (Fig. 1G,H). GSE172002 and GSE236654 shared 141 DEGs, including 41 up-regulated genes and 100 down-regulated genes (Fig. 1I). Overlay with the WikiPathways Cytosolic DNA-sensing pathway (WP4655) identified cGAS and CXCL10 as shared members, supporting the rationale for further examining cGAS in this context (Fig. 1J). Given that CXCL10 functions downstream and chiefly mediates immune-cell recruitment, its relevance to tumor cell–autonomous resistance is limited in our models. We thus prioritized cGAS for mechanistic investigation.

Subsequent validation showed that EGFR-TKI treatment increased cGAS protein and mRNA levels in parental H1975 and PC-9 cells, while cGAS induction was weaker in resistant cells (Fig. 1K,L). Immunofluorescence staining showed a similar trend, with enhanced cGAS signals after EGFR-TKI exposure in parental cells and relatively lower changes in H1975/OR and PC-9/GR cells (Fig. 1M).

Together, these results indicate that EGFR-TKI treatment is associated with DNA damage, cytosolic dsDNA accumulation, and cGAS activation, particularly in EGFR-TKI-sensitive NSCLC cells. Resistant cells showed reduced inhibition of EGFR downstream signaling and a comparatively attenuated DNA damage/cGAS response, suggesting that altered DNA damage-related stress responses may be linked to EGFR-TKI resistance.

### 3.2 Differential Ferroptotic Responses to EGFR-TKIs in Sensitive and Drug-Resistant NSCLC Cells

**Figure 2:**
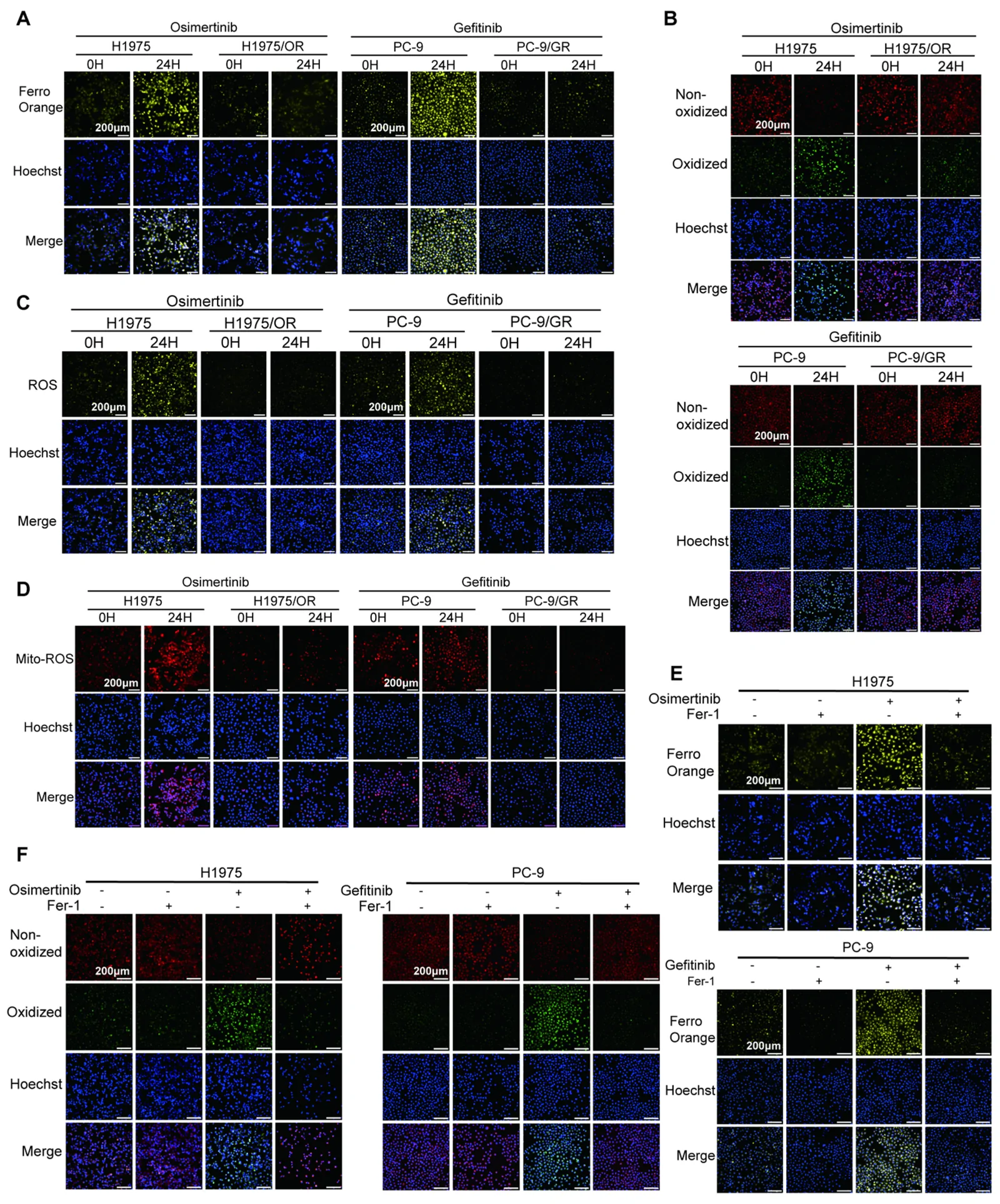
Differential ferroptotic responses to EGFR-TKIs in sensitive and drug-resistant NSCLC cells. (**A**) Representative FerroOrange staining in EGFR-TKI–sensitive (PC-9, H1975) and resistant (PC-9/GR, H1975/OR) cells following gefitinib or osimertinib treatment (scale bar = 200 μm). (**B**) Representative C11 BODIPY 581/591 staining in sensitive and resistant cells after EGFR-TKI exposure (scale bar = 200 μm). (**C**) Representative CellROX Orange staining showing intracellular ROS-associated fluorescence signals (scale bar = 200 μm). (**D**) Representative MitoSOX Red staining showing mitochondrial ROS-associated fluorescence signals (scale bar = 200 μm). (**E**) FerroOrange staining showing that ferroptosis inhibitor Ferrostatin-1 (Fer-1) attenuated osimertinib- or gefitinib-induced Fe^2^^+^ accumulation in H1975 and PC-9 cells (scale bar = 200 μm). (**F**) C11 BODIPY 581/591 staining showing that Fer-1 reduced EGFR-TKI-induced lipid peroxidation in H1975 and PC-9 cells (scale bar = 200 μm). Hoechst was used for nuclear counterstaining.

Previous studies have demonstrated that ferroptosis is closely related to the progression of NSCLC and chemotherapy resistance [19]. To determine whether EGFR-TKI treatment was accompanied by ferroptosis-related changes, we first evaluated intracellular Fe^2^^+^ accumulation using FerroOrange staining. After 24 h of osimertinib treatment, H1975 cells showed an increase in FerroOrange fluorescence, whereas this response was less apparent in H1975/OR cells. Similarly, gefitinib treatment increased FerroOrange signals in PC-9 cells, while PC-9/GR cells displayed a comparatively weaker change under the same treatment condition (Fig. 2A). The divergence in FerroOrange intensity indicated that labile Fe^2^^+^ increased preferentially in the parental populations after matched drug exposure.

We next examined lipid peroxidation using C11 BODIPY 581/591 staining. In H1975 and PC-9 cells, EGFR-TKI exposure was accompanied by increased oxidized lipid signals, reflected by enhanced green fluorescence and reduced non-oxidized red fluorescence. In contrast, H1975/OR and PC-9/GR cells showed a relatively attenuated lipid oxidation pattern after osimertinib or gefitinib treatment (Fig. 2B). Consistently, intracellular ROS and mitochondrial ROS signals were increased in parental H1975 and PC-9 cells following EGFR-TKI treatment, whereas these changes were less evident in the corresponding resistant cells (Fig. 2C,D). The concurrent rise in oxidized C11-BODIPY, CellROX, and MitoSOX signals therefore localized the stronger oxidative response to the drug-sensitive cells.

To further assess mitochondrial functional changes, JC-1 staining was performed. JC-1 staining showed a shift toward weaker aggregate-associated red fluorescence and relatively stronger monomer-associated green fluorescence in EGFR-TKI-treated parental cells, whereas this staining pattern was less evident in resistant cells (Supplementary Fig. S2A). In line with these observations, EGFR-TKI treatment decreased GSH levels and increased MDA levels in parental H1975 and PC-9 cells, while these changes were less pronounced in resistant cells (Supplementary Fig. S2B). Together, these results suggest that EGFR-TKI-sensitive cells exhibit more evident ferroptosis-associated oxidative changes and mitochondrial dysfunction-related features than resistant cells.

To clarify whether these changes were related to ferroptosis, cells were treated with the ferroptosis inhibitor Ferrostatin-1 (Fer-1). Fer-1 attenuated EGFR-TKI-associated FerroOrange fluorescence in H1975 and PC-9 cells (Fig. 2E). Similarly, Fer-1 reduced the EGFR-TKI-associated increase in oxidized C11 BODIPY signals induced by osimertinib or gefitinib (Fig. 2F). In parallel, Fer-1 also reduced intracellular ROS accumulation and partially restored GSH levels while decreasing MDA accumulation in EGFR-TKI-treated parental cells (Supplementary Fig. S2C–E). Suppression of these endpoints by Fer-1 indicates that a ferroptotic component contributed to the drug-induced redox phenotype, although it may not account for the entire response.

Collectively, these data indicate that EGFR-TKI treatment was associated with fluorescence patterns consistent with Fe^2^^+^ accumulation, lipid peroxidation, intracellular ROS, and mitochondrial ROS changes, together with biochemical evidence of GSH reduction and MDA increase. Compared with parental cells, drug-resistant cells showed a relatively attenuated ferroptosis-associated response, suggesting that reduced susceptibility to ferroptotic oxidative damage may be involved in the resistant phenotype.

### 3.3 Involvement of cGAS in the Tumor Growth of EGFR-TKI-Sensitive and -Resistant NSCLC

**Figure 3:**
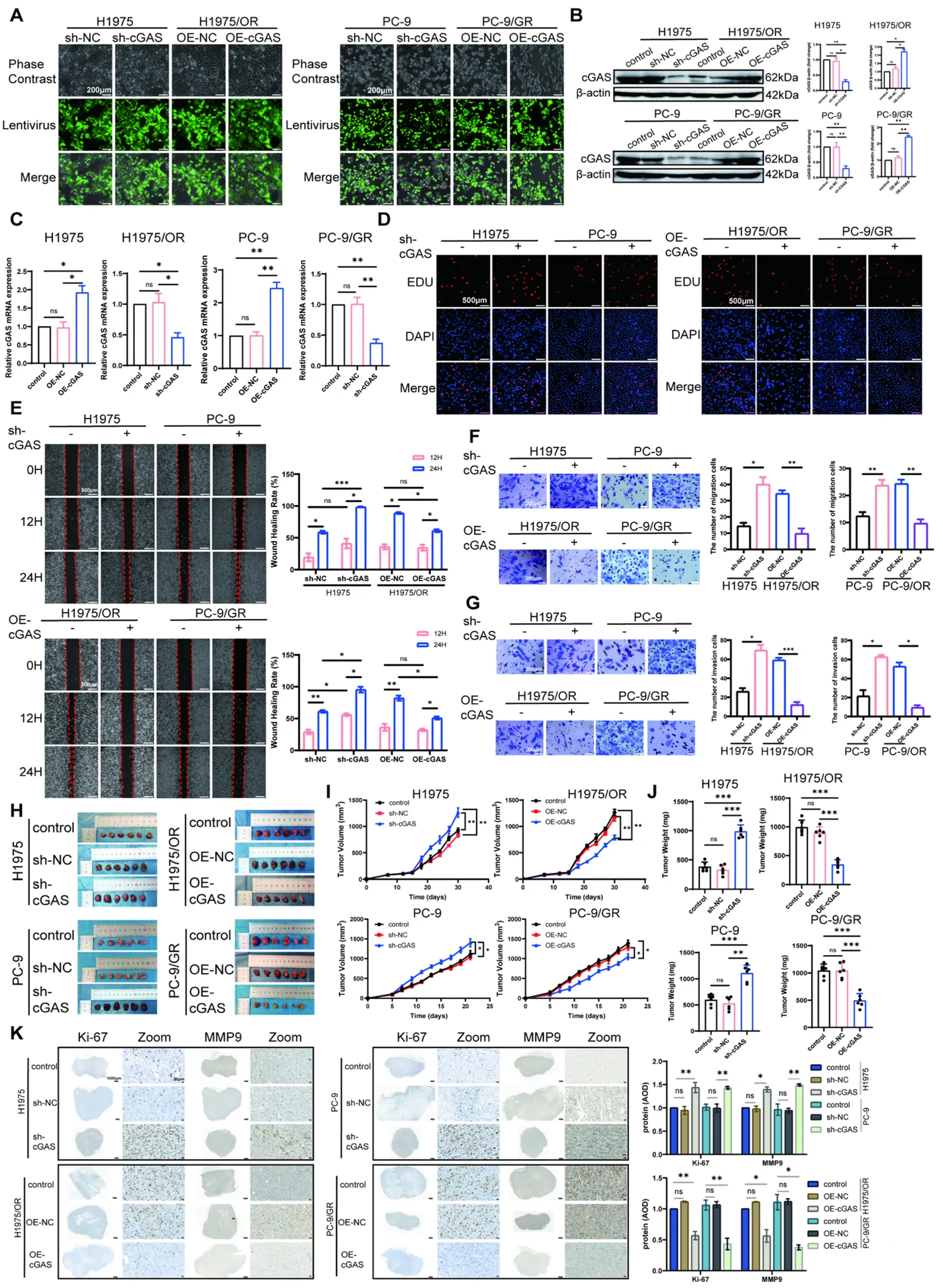
Involvement of cGAS in the tumor growth of EGFR-TKI-sensitive and -resistant NSCLC. (**A**) Representative fluorescence images showing lentiviral transduction efficiency of sh-cGAS, OE-cGAS in PC-9, H1975, PC-9/GR, and H1975/OR cells (scale bar = 200 μm). (**B**) Western blot analysis of cGAS protein levels in transduced cells. (**C**) qRT-PCR analysis of cGAS mRNA levels in transduced cells. (**D**) EdU incorporation assay assessing cell proliferation (scale bar = 500 μm). (**E**) Wound healing assay assessing cell migration (scale bar = 500 μm). (**F**) Transwell migration assay assessing migratory capacity (scale bar = 200 μm). (**G**) Transwell invasion assay assessing invasive capacity (scale bar = 200 μm). (**H**) Representative xenograft tumors from nude mice implanted with transduced cell lines. (**I**) Tumor growth curves of xenografts derived from transduced cell lines. (**J**) Endpoint tumor weights of xenografts derived from transduced cell lines. (**K**) Immunohistochemical staining for Ki-67 and MMP-9 in xenograft sections and semi-quantitative analysis of average optical density (AOD) (scale bars = 1000 μm and 20 μm). *p < 0.05, **p < 0.01, ***p < 0.001, *ns*, not significant.

To determine how cGAS influences malignant behavior under different EGFR-TKI response states, we generated cGAS-deficient H1975 and PC-9 cells and established cGAS-overexpressing H1975/OR and PC-9/GR cells. Successful lentiviral delivery was verified by fluorescence microscopy, and the corresponding changes in cGAS abundance were confirmed at both the protein and transcript levels (Fig. 3A–C).

DNA synthesis was then assessed by EdU incorporation. Loss of cGAS increased the proportion of EdU-positive cells in the parental H1975 and PC-9 populations. By contrast, forced expression of cGAS reduced EdU labeling in H1975/OR and PC-9/GR cells (Fig. 3D). EdU incorporation changed in the opposite direction to cGAS abundance, linking low cGAS to increased DNA synthesis in the examined models.

We next examined whether cGAS also affected cell movement. In scratch assays, depletion of cGAS accelerated closure of the wound area in parental cells, whereas cGAS restoration delayed closure in the resistant derivatives (Fig. 3E). The Transwell experiments yielded a concordant pattern: cGAS-silenced H1975 and PC-9 cells exhibited greater migratory and invasive activity, while fewer H1975/OR and PC-9/GR cells traversed the membrane after cGAS overexpression (Fig. 3F,G). The scratch and Transwell measurements converged on the same pattern: cGAS loss enhanced motility in parental cells, while its restoration restricted migration and invasion in resistant derivatives.

The biological relevance of these observations was further evaluated in nude-mouse xenografts established from the corresponding modified cell lines. Tumors originating from cGAS-knockdown H1975 or PC-9 cells expanded more rapidly and reached greater final volumes and weights than their matched controls. The opposite pattern was observed in xenografts derived from cGAS-overexpressing H1975/OR and PC-9/GR cells, which displayed slower enlargement and lower tumor mass (Fig. 3H–J). The *in vivo* growth behavior was therefore consistent with the proliferation and motility phenotypes observed in culture.

Immunohistochemical evaluation provided additional support for these findings. Xenografts formed by cGAS-deficient cells showed stronger Ki-67 and MMP9 staining, whereas resistant-cell tumors with restored cGAS exhibited lower expression of both markers (Fig. 3K). Overall, these data identify cGAS as a negative regulator of tumor growth and aggressive cellular behavior in the examined NSCLC models. Its reduction promoted proliferation, migration, invasion, and xenograft expansion, while its re-expression partially suppressed these features in EGFR-TKI-resistant cells.

### 3.4 cGAS Modulates Ferroptosis Susceptibility and ROS Homeostasis in EGFR-TKI-Sensitive and -Resistant NSCLC Cells

**Figure 4:**
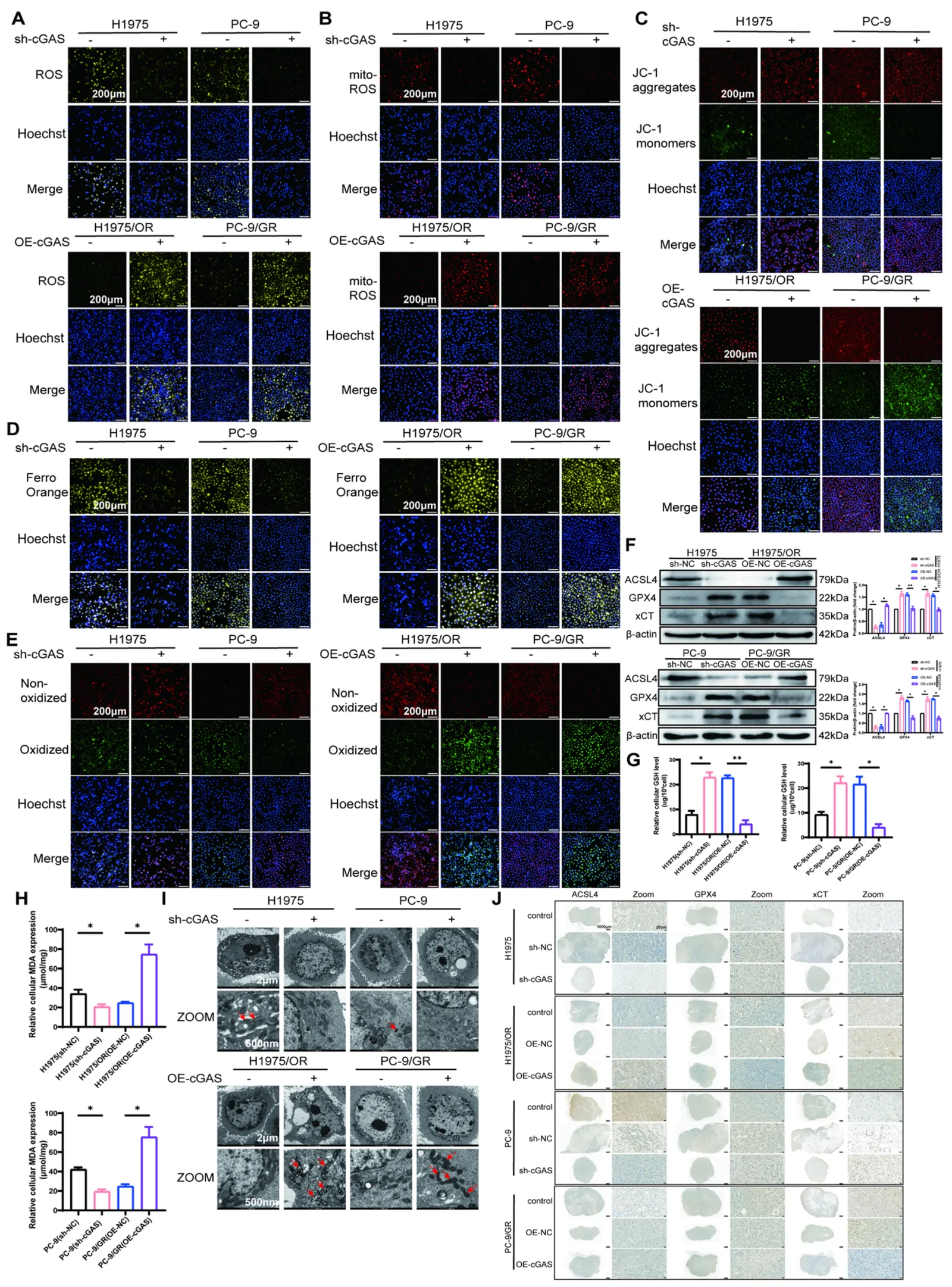
cGAS modulates ferroptosis susceptibility and ROS homeostasis in EGFR-TKI-sensitive and -resistant NSCLC cells. (**A**) CellROX fluorescence imaging of intracellular reactive oxygen species in H1975 and PC-9 cells after cGAS knockdown and in H1975/OR and PC-9/GR cells after cGAS overexpression (scale bar = 200 μm). Hoechst staining identifies nuclei. (**B**) Representative MitoSOX™ staining showing mitochondrial ROS-associated fluorescence signals in sh-cGAS or OE-cGAS NSCLC cells (scale bar = 200 μm). (**C**) Representative JC-1 staining showing mitochondrial membrane potential-related staining patterns in sh-cGAS or OE-cGAS NSCLC cells (scale bar = 200 μm). (**D**) Representative FerroOrange staining showing Fe^2^^+^-associated fluorescence signals in sh-cGAS or OE-cGAS NSCLC cells (scale bar = 200 μm). (**E**) Representative C11-BODIPY™ staining showing lipid peroxidation-associated fluorescence signals in sh-cGAS or OE-cGAS NSCLC cells (scale bar = 200 μm). (**F**) WB analysis of ferroptosis-related proteins GPX4, xCT, and ACSL4. (**G**) Quantification of intracellular GSH levels. (**H**) Quantification of intracellular MDA levels. (**I**) Transmission electron microscopy of mitochondrial ultrastructure. Red arrows indicate representative mitochondria with altered ultrastructural morphology (scale bars = 2 μm, 500 nm). (**J**) IHC analysis of GPX4, xCT, and ACSL4 expression in xenograft tumors derived from PC-9, H1975, PC-9/GR, and H1975/OR cells with indicated cGAS modulation (scale bars = 1000 μm and 20 μm). *p < 0.05, **p < 0.01.

cGAS, as a cytoplasmic DNA sensor, not only participates in the innate immune response but also potentially influences tumor cell growth by regulating DNA damage repair and ferroptosis [14]. To evaluate whether cGAS is involved in ferroptosis-related redox regulation, we first examined intracellular ROS and mitochondrial ROS after cGAS modulation. Depletion of cGAS lowered both CellROX and MitoSOX intensity in parental cells; restoring cGAS in the resistant derivatives increased the two signals (Fig. 4A,B). JC-1 staining further showed that cGAS knockdown tended to preserve mitochondrial membrane potential in parental cells, whereas cGAS overexpression in resistant cells was accompanied by reduced JC-1 aggregates and increased JC-1 monomers, suggesting impaired mitochondrial status (Fig. 4C).

We next assessed ferroptosis-associated changes. cGAS depletion decreased FerroOrange intensity and the oxidized C11-BODIPY signal, while cGAS restoration in resistant cells increased both readouts (Fig. 4D,E). Western blot analysis further showed that cGAS knockdown was associated with lower ACSL4 and higher GPX4 and xCT expression, whereas cGAS overexpression showed the opposite tendency in H1975/OR and PC-9/GR cells (Fig. 4F).

Biochemical assays were generally consistent with the imaging and protein data. cGAS knockdown increased GSH levels and reduced MDA accumulation in parental cells, while cGAS overexpression decreased GSH levels and increased MDA levels in resistant cells (Fig. 4G,H). Similar trends were observed in xenograft tumor tissues, where cGAS knockdown was associated with higher GSH and lower MDA levels, whereas cGAS overexpression showed lower GSH and higher MDA levels (Supplementary Fig. S3A,B). Transmission electron microscopy also showed fewer ferroptosis-like mitochondrial alterations after cGAS knockdown, while cGAS overexpression was accompanied by more apparent mitochondrial structural abnormalities in resistant cells (Fig. 4I). In xenograft tissues, immunohistochemical staining of ACSL4, GPX4, and xCT further supported these ferroptosis-related changes (Fig. 4J).

To further examine whether the effects associated with cGAS overexpression were linked to ferroptosis, resistant cells were treated with Ferrostatin-1 (Fer-1). In PC-9/GR and H1975/OR cells, cGAS overexpression was associated with fluorescence patterns consistent with increased Fe^2^^+^-related signals, lipid peroxidation-associated signals, and ROS-associated signals, particularly under gefitinib or osimertinib exposure (Supplementary Fig. S4A–C). Fer-1 also partially restored GSH levels and reduced MDA accumulation in cGAS-overexpressing resistant cells (Supplementary Fig. S4D,E).

Together, these results suggest that cGAS is associated with ROS homeostasis and ferroptosis susceptibility in NSCLC cells. In parental cells, cGAS knockdown showed a relatively protective redox pattern, whereas in EGFR-TKI-resistant cells, cGAS overexpression was accompanied by enhanced ferroptosis-related oxidative injury. The partial reversal by Fer-1 further supports that these cGAS-associated changes are, at least in part, related to ferroptotic responses.

### 3.5 SIRT3 Is Implicated in the Ferroptotic Response to EGFR-TKIs in NSCLC

**Figure 5:**
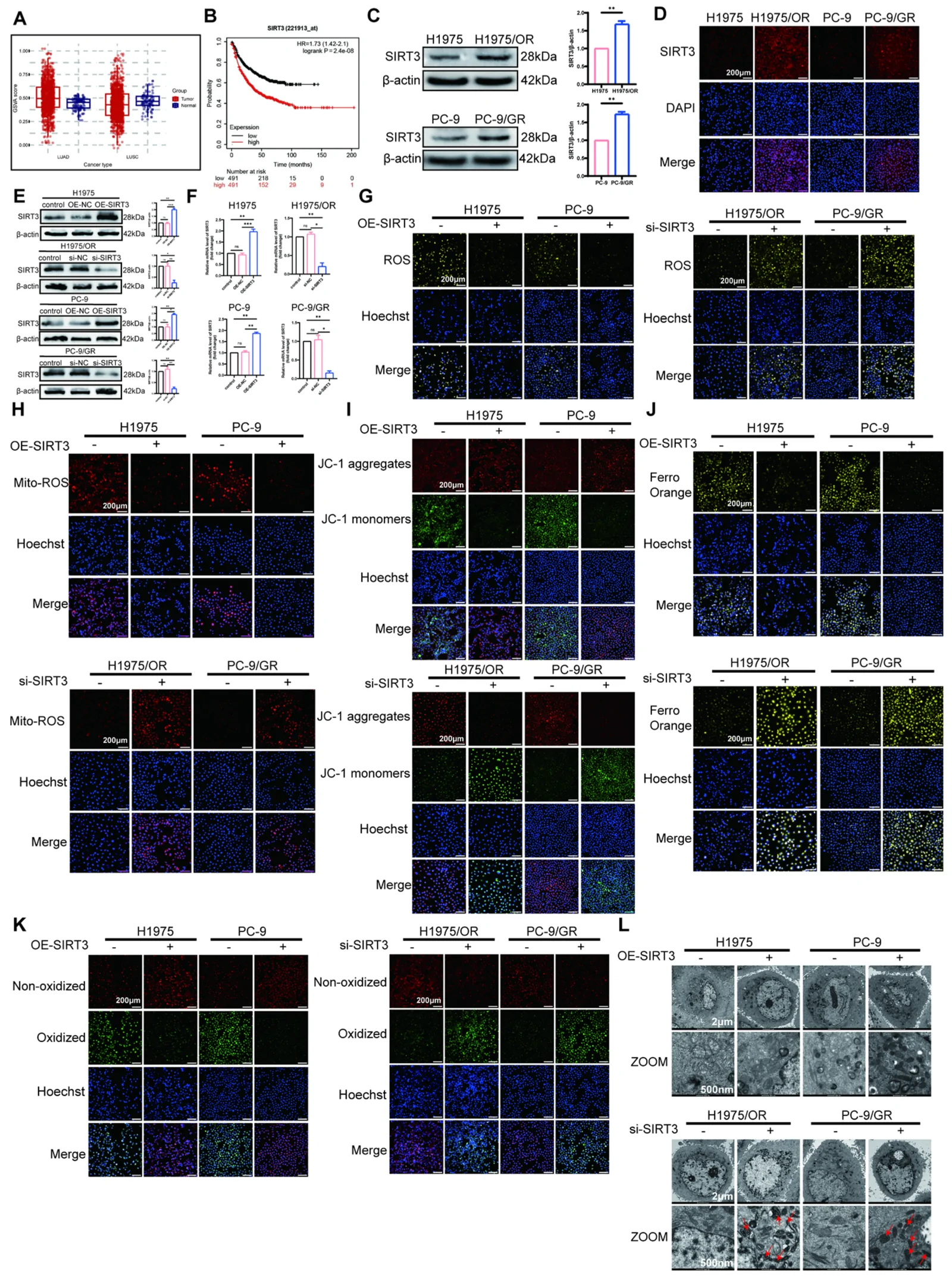
SIRT3 is implicated in the ferroptotic response to EGFR-TKIs in NSCLC. (**A**) GSCA analysis of SIRT3 expression in LUAD, LUSC, and normal lung tissues. (**B**) Kaplan–Meier survival curves stratified by SIRT3 expression. (**C**) Western blot analysis of SIRT3 expression. (**D**) Immunofluorescence staining of SIRT3 expression (scale bar = 100 μm). (**E**) Western blot validation of SIRT3 knockdown or overexpression efficiency. (**F**) qRT-PCR validation of SIRT3 knockdown or overexpression efficiency. (**G**) Representative CellROX staining after SIRT3 modulation (scale bar = 200 μm). (**H**) Representative MitoSOX staining after SIRT3 modulation (scale bar = 200 μm). (**I**) Representative JC-1 staining after SIRT3 modulation (scale bar = 200 μm). (**J**) Representative FerroOrange staining after SIRT3 modulation (scale bar = 200 μm). (**K**) Representative C11-BODIPY staining after SIRT3 modulation (scale bar = 200 μm). (**L**) TEM images showing mitochondrial ultrastructure. Red arrows indicate representative mitochondria with altered ultrastructural morphology (scale bars = 2 μm, 500 nm). *p < 0.05, **p < 0.01, ***p < 0.001, *ns*, not significant.

Previous studies [27] have suggested a functional relationship between cGAS signaling and SIRT3-mediated mitochondrial regulation. Therefore, we next examined whether SIRT3 was involved in the ferroptosis-related phenotype of EGFR-TKI-resistant NSCLC cells. SIRT3 is an NAD^+^-dependent deacetylase whose functions include maintaining the integrity of mitochondrial membranes and reducing the generation of ROS [28]. Research has indicated that there is a mutual regulatory relationship between cGAS and SIRT3, which participates in the regulation of ROS [27]. Bioinformatic analysis using the GSCA platform indicated that SIRT3 expression was elevated in both lung adenocarcinoma and lung squamous cell carcinoma (Fig. 5A). Kaplan–Meier survival analysis further showed that high SIRT3 expression correlated with poor prognosis in lung cancer patients (Fig. 5B). Consistently, WB and immunofluorescence assays demonstrated that SIRT3 protein levels were markedly higher in EGFR-TKI–resistant PC-9/GR and H1975/OR cells compared with their parental lines (Fig. 5C,D).

To assess its functional role, SIRT3 was silenced in resistant cells via siRNA and overexpressed in sensitive cells via plasmid transfection, with successful modulation confirmed by WB and qRT–PCR (Fig. 5E,F). Under EGFR-TKI treatment, representative staining images showed that SIRT3 overexpression in sensitive cells was associated with weaker intracellular and mitochondrial ROS-associated fluorescence and a more preserved JC-1 staining pattern. Conversely, SIRT3 knockdown in resistant cells was associated with stronger ROS-associated fluorescence and an altered JC-1 staining pattern (Fig. 5G–I). FerroOrange and C11-BODIPY staining showed attenuated Fe^2^^+^- and lipid peroxidation-associated fluorescence patterns after SIRT3 overexpression, whereas opposite staining patterns were observed after SIRT3 knockdown (Fig. 5J,K). Transmission electron microscopy showed that mitochondria in resistant cells remained structurally intact after EGFR-TKI exposure, but SIRT3 knockdown induced ferroptosis-like mitochondrial changes, including cristae loss and membrane condensation. In sensitive cells, EGFR-TKI–induced mitochondrial damage was alleviated by SIRT3 overexpression (Fig. 5L).

Together, the gain- and loss-of-function results place SIRT3 among the factors that protect resistant cells from mitochondrial oxidative damage and ferroptosis-associated alterations.

### 3.6 cGAS Modulates Ferroptosis Potentially via the p-Nrf2-SIRT3 Pathway

**Figure 6:**
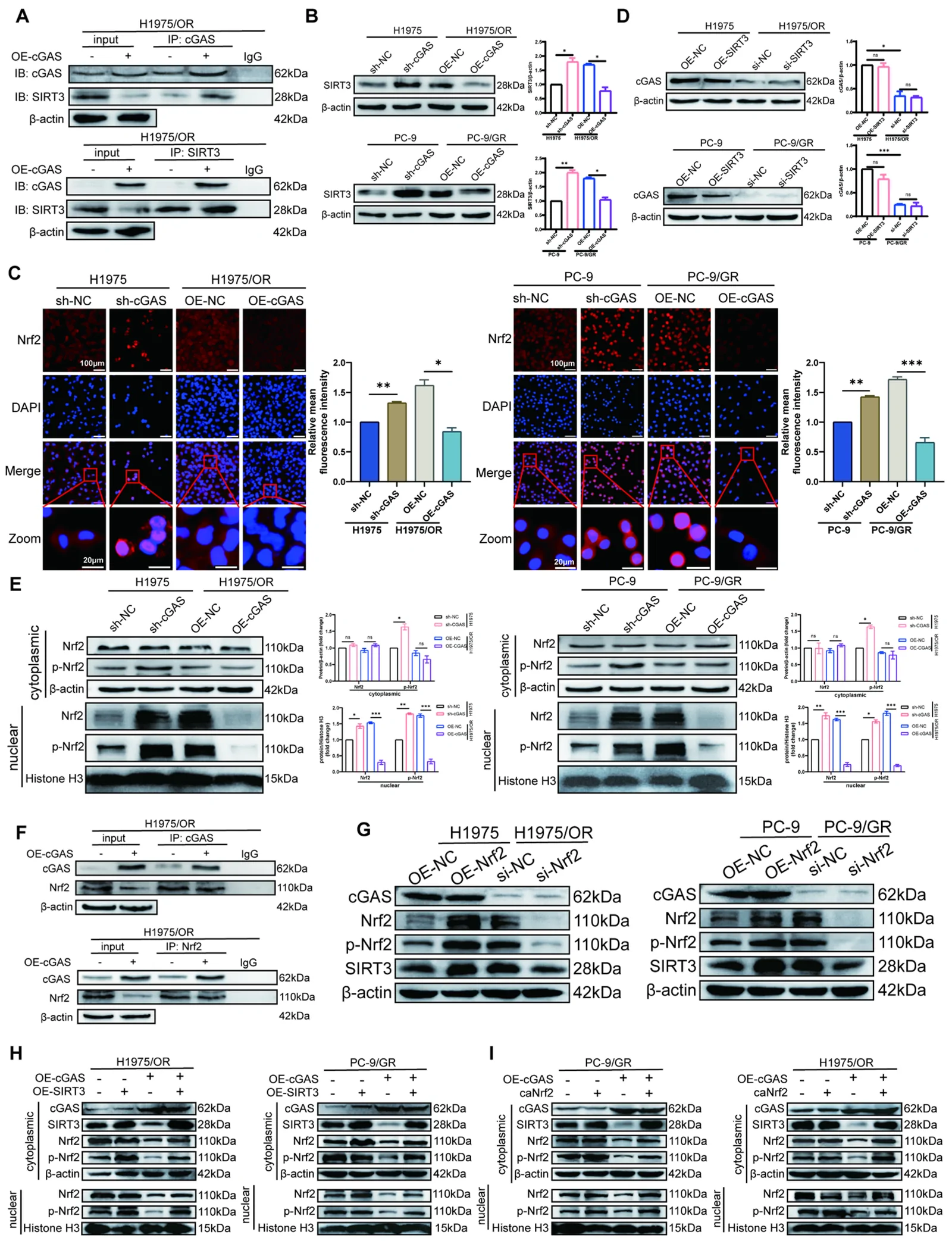
cGAS modulates ferroptosis potentially via the p-Nrf2-SIRT3 pathway. (**A**) Reciprocal co-immunoprecipitation analysis of cGAS and SIRT3 in H1975/OR cells with or without cGAS overexpression. (**B**) Western blot analysis of SIRT3 expression after cGAS knockdown in H1975 and PC-9 cells or cGAS overexpression in H1975/OR and PC-9/GR cells. (**C**) Representative immunofluorescence images and quantification of Nrf2 staining in the indicated cell groups (scale bars = 100 μm, 20 μm). (**D**) Western blotting and densitometric analysis of cGAS after SIRT3 overexpression in parental cells or SIRT3 knockdown in resistant cells. (**E**) Cytoplasmic and nuclear fractionation followed by western blotting to detect Nrf2 and p-Nrf2 levels after cGAS modulation. (**F**) Reciprocal co-immunoprecipitation analysis of cGAS and Nrf2 in H1975/OR cells with or without cGAS overexpression. (**G**) Western blotting of cGAS, Nrf2, p-Nrf2 (Ser40), and SIRT3 after Nrf2 overexpression in parental cells or Nrf2 knockdown in resistant cells. (**H**) Cytoplasmic and nuclear levels of cGAS, SIRT3, Nrf2, and p-Nrf2 (Ser40) after cGAS overexpression alone or combined with SIRT3 overexpression in H1975/OR and PC-9/GR cells. (**I**) Cytoplasmic and nuclear levels of cGAS, SIRT3, Nrf2, and p-Nrf2 (Ser40) after cGAS overexpression alone or combined with constitutively active Nrf2 in H1975/OR and PC-9/GR cells. β-actin and Histone H3 were used as cytoplasmic and nuclear loading controls, respectively. Data are presented as mean ± SD. *p < 0.05, **p < 0.01, ***p < 0.001; *ns*, not significant. p-Nrf2 indicates Nrf2 phosphorylated at Ser40.

To explore how cGAS may participate in ferroptosis-related regulation, we examined its relationship with the Nrf2/SIRT3 pathway in EGFR-TKI-sensitive and -resistant NSCLC cells. Co-immunoprecipitation assays showed a detectable association between cGAS and SIRT3 in H1975/OR cells (Fig. 6A). Consistent with these observations, cGAS knockdown in H1975 and PC-9 cells was associated with increased SIRT3 protein levels, mRNA expression, and enzymatic activity. Conversely, cGAS overexpression in H1975/OR and PC-9/GR cells reduced SIRT3 expression and activity (Fig. 6B; Supplementary Fig. S5A,B). The reciprocal changes in SIRT3 abundance and activity position SIRT3 downstream of cGAS-dependent regulation in these models.

Because Nrf2 is an important transcription factor involved in redox homeostasis and has been reported to promote SIRT3 expression, we further assessed whether cGAS was related to Nrf2 expression. Immunofluorescence staining showed that cGAS knockdown increased Nrf2 fluorescence, with a more evident nuclear accumulation pattern in H1975 and PC-9 cells. In contrast, cGAS overexpression reduced Nrf2 staining in H1975/OR and PC-9/GR cells (Fig. 6C). Nuclear and cytoplasmic fractionation further showed that cGAS modulation was associated with changes in both Nrf2 and p-Nrf2 levels, particularly in the nuclear fraction (Fig. 6E). cGAS manipulation therefore altered three separable features of Nrf2 regulation: total abundance, Ser40 phosphorylation, and nuclear partitioning.

Additional analyses supported a possible association between cGAS and Nrf2. Co-immunoprecipitation detected an association between cGAS and Nrf2 in H1975/OR cells (Fig. 6F). Predicted structural modeling and molecular docking analysis also suggested a potential spatial interaction between cGAS and Nrf2 (Supplementary Fig. S5C,D). The co-IP and docking findings support molecular proximity between cGAS and Nrf2 but do not establish direct binding or define the biochemical consequence of the association.

We next examined whether Nrf2 was linked to SIRT3 expression in this system. Nrf2 overexpression increased SIRT3 protein expression, mRNA expression, and enzymatic activity in H1975 and PC-9 cells, whereas Nrf2 knockdown reduced SIRT3 expression and activity in H1975/OR and PC-9/GR cells (Fig. 6G; Supplementary Fig. S5F–H). In contrast, SIRT3 modulation did not consistently alter cGAS mRNA expression, although changes in cGAS protein were observed in some conditions (Fig. 6D; Supplementary Fig. S5E). These results suggest that Nrf2 may act as an upstream regulator of SIRT3, while the relationship between SIRT3 and cGAS may not be explained solely by transcriptional regulation.

Finally, rescue experiments were performed to further assess the pathway relationship. In resistant H1975/OR and PC-9/GR cells, SIRT3 overexpression partially modified the Nrf2/p-Nrf2 nuclear distribution pattern observed under cGAS-overexpressing conditions, especially in the nuclear fraction (Fig. 6H; Supplementary Fig. S5I). Similarly, constitutively active Nrf2 partially restored SIRT3 expression and nuclear Nrf2/p-Nrf2 abundance under cGAS-overexpressing conditions (Fig. 6I; Supplementary Fig. S5J).

Across the fractionation, expression, activity, and rescue experiments, altering cGAS consistently changed nuclear p-Nrf2 availability and the SIRT3 functional state. In combination with the ferroptosis-related results, the data support a cautious model in which cGAS may modulate ferroptosis susceptibility, at least in part, through the p-Nrf2/SIRT3 pathway in EGFR-TKI-sensitive and -resistant NSCLC cells.

## 4 Discussion

EGFR-TKIs have substantially improved the treatment of EGFR-mutant NSCLC, but acquired resistance remains a major clinical challenge [29,30]. Established mechanisms, including secondary EGFR mutations, bypass signaling, histologic transformation, and epigenetic remodeling, explain many cases; however, a proportion of resistant tumors remain incompletely characterized [3,31,32]. Our previous studies point to coordinated adaptation across genomic-stress sensing, antioxidant control, and ferroptotic tolerance as another layer of the resistant phenotype [33,34,35]. In this context, the present study focused on the role of cGAS in EGFR-TKI-resistant NSCLC, particularly its association with redox regulation and ferroptosis-related vulnerability.

Transcriptomic analysis showed significant alteration of the HALLMARK_DNA_REPAIR pathway in both datasets, although the enrichment direction differed between GSE172002 and GSE236654, supporting a potential relationship between DNA repair-related programs and EGFR-TKI resistance. In cell models, parental PC-9 and H1975 cells showed marked suppression of p-EGFR, p-AKT1, and p-ERK1/2 after gefitinib or osimertinib treatment, whereas the corresponding resistant derivatives displayed attenuated signaling inhibition. EGFR-TKI exposure was also associated with more evident DNA damage in parental cells, as reflected by comet assay, γ-H2AX staining, and DNA repair-related marker changes. BRCA1/RAD51 and Ku70/Ku80 were measured as indicators of repair-related responses; without pathway-specific reporter assays, their changes cannot establish a functional shift between homologous recombination and non-homologous end joining [36,37].

Consistent with this DNA damage-related pattern, EGFR-TKI-sensitive cells showed greater cytosolic dsDNA accumulation and stronger cGAS induction after drug exposure, whereas resistant cells displayed a more limited response. These findings raise the possibility that altered DNA damage handling in resistant cells may reduce cytosolic DNA-associated stress signaling. However, this interpretation remains inferential, because functional NHEJ activity, cGAMP production, and downstream cGAS-STING markers such as STING/TBK1 activation were not directly measured [38,39]. The data therefore establish correlation across these events, while the causal sequence from DNA repair activity to cytosolic DNA sensing remains to be tested experimentally.

cGAS exerted a consistent growth-restrictive effect across the two cellular states examined. In parental EGFR-TKI-sensitive cells, cGAS knockdown was associated with increased proliferation, migration, invasion, and xenograft growth-related features. Conversely, in resistant cells, cGAS restoration suppressed these malignant phenotypes and reduced tumor growth *in vivo*. These findings support the interpretation that reduced cGAS expression is linked to the resistant phenotype. Because the xenograft experiments did not include matched gefitinib or osimertinib treatment arms, the *in vivo* data should be interpreted as evidence that cGAS modulation affects resistant tumor growth rather than as direct proof of *in vivo* EGFR-TKI resensitization. The present data also suggest that cGAS is associated with ferroptosis-related redox regulation. EGFR-TKI-sensitive cells showed more evident staining patterns associated with Fe^2^^+^ accumulation, lipid peroxidation, ROS generation, and mitochondrial dysfunction, together with lower GSH and higher MDA after drug exposure. cGAS knockdown in parental cells was accompanied by a more ferroptosis-resistant redox pattern, while cGAS overexpression in resistant cells showed opposite staining patterns, including increased ROS, Fe^2^^+^ accumulation, lipid peroxidation, ACSL4 expression, and MDA levels, together with lower GPX4/xCT expression and GSH. These findings were further supported by TEM observations, IHC staining, and GSH/MDA measurements in xenograft tissues. The incomplete rescue achieved with Ferrostatin-1 indicates that ferroptotic injury accounts for part, but probably not all, of the phenotype produced by cGAS manipulation.

SIRT3 appeared to be an important component of this redox-adaptive program. Resistant cells expressed higher SIRT3 levels than parental cells, and SIRT3 overexpression in sensitive cells was associated with reduced ROS accumulation, improved mitochondrial membrane potential, and weaker ferroptosis-related changes after EGFR-TKI treatment. Conversely, SIRT3 knockdown in resistant cells increased oxidative stress, impaired mitochondrial status, and enhanced ferroptosis-associated features. These findings are consistent with the known role of SIRT3 in mitochondrial homeostasis and antioxidant regulation [40,41,42], and suggest that SIRT3 may help buffer oxidative stress in the resistant state.

**Figure 7:**
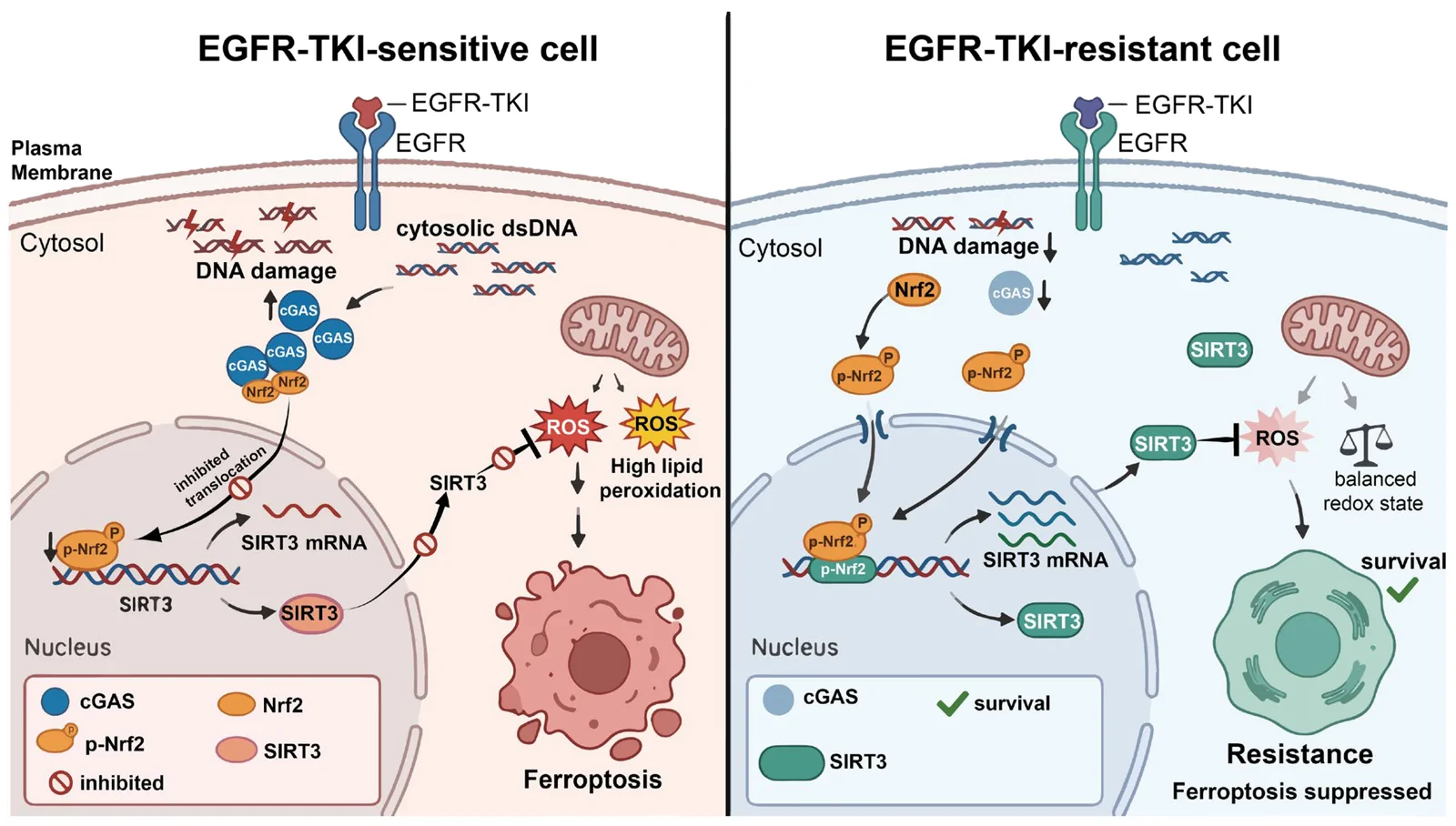
Mechanistic diagram of proposed model by which cGAS may influence EGFR-TKI resistance via ferroptosis regulation through the p-Nrf2-SIRT3-ROS axis. **EGFR-TKI-sensitive cells:** Following EGFR-TKI treatment, NSCLC cells display increased cytosolic and nuclear DNA damage, accompanied by upregulation of cGAS. Enhanced cGAS–Nrf2 association may be associated with reduced p-Nrf2 nuclear availability and impedes Nrf2 nuclear translocation, thereby reducing SIRT3 expression, elevating ROS, and promoting ferroptosis. **EGFR-TKI–resistant cells:** Upon EGFR-TKI treatment, resistant NSCLC cells exhibit less EGFR-TKI-induced DNA damage. cGAS expression and association with Nrf2 are diminished, whereas p-Nrf2 and its nuclear accumulation are increased. Consequently, SIRT3 expression is upregulated, ROS generation is attenuated, and the ferroptotic program is suppressed.

A central finding of this study is a potential link between cGAS and the Nrf2–SIRT3 antioxidant axis. Nrf2 has been reported to promote SIRT3 transcription and expression [43], and our data support a relationship between Nrf2 activity and SIRT3 expression in EGFR-TKI-sensitive and -resistant NSCLC cells. Nrf2 overexpression increased SIRT3 mRNA, protein expression, and enzymatic activity, whereas Nrf2 knockdown reduced them. In contrast, Nrf2 manipulation did not obviously alter cGAS expression, and SIRT3 modulation did not consistently change cGAS mRNA levels. These observations support the possibility that cGAS acts upstream of the Nrf2–SIRT3 axis, while SIRT3 functions as a downstream redox-regulatory component (Fig. 7).

The relationship between cGAS and Nrf2 should be interpreted with caution. Co-immunoprecipitation supported an association between cGAS and Nrf2, and nuclear/cytoplasmic fractionation showed that cGAS knockdown increased, whereas cGAS overexpression reduced, the nuclear accumulation of total Nrf2 and p-Nrf2. These results are consistent with the possibility that cGAS may, at least in part, limit the nuclear availability of p-Nrf2 and thereby reduce SIRT3 transcription. However, the current data do not fully distinguish whether cGAS mainly affects p-Nrf2 nuclear trafficking, upstream phosphorylation, or phosphatase-mediated dephosphorylation. Accordingly, the present experiments define the direction of association but do not yet distinguish altered phosphorylation from changes in nuclear transport or dephosphorylation. Similarly, the cGAS–SIRT3 co-IP signal is more appropriately interpreted as co-complex formation or association rather than direct protein–protein binding.

The docking analysis provides only preliminary structural support for this model. Because the docking was based on a total Nrf2 model rather than a phospho-specific Nrf2 conformation, the predicted interface should be viewed as a computational hypothesis for a possible cGAS–Nrf2 association rather than direct structural evidence for a phosphorylation-state-specific interaction. Additional biophysical binding studies will be needed to validate the proposed interface and define its binding properties more rigorously.

The possible involvement of canonical cGAS-STING signaling also deserves consideration. cGAS is classically recognized as a cytosolic DNA sensor that can activate STING-dependent innate immune signaling, and its biological consequences in cancer are context-dependent [44,45]. Here, we focused on the tumor cell-intrinsic role of cGAS in EGFR-TKI-resistant cells, especially its association with the p-Nrf2/SIRT3/redox-ferroptosis framework. Because STING expression and activation were not directly examined, we cannot exclude the possibility that STING-dependent signaling contributes to part of the observed phenotype. Therefore, our findings support a cGAS-associated redox-ferroptosis regulatory model rather than excluding canonical cGAS-STING involvement.

The potential novelty of this work lies in placing cGAS-associated ferroptosis regulation within the specific context of EGFR-TKI-resistant NSCLC. Rather than describing cGAS or ferroptosis as isolated pathways, this study suggests that reduced cGAS expression may be linked to resistant-state adaptation through altered DNA damage-related stress responses, Nrf2/SIRT3-associated antioxidant buffering, and reduced ferroptosis susceptibility. This context-specific perspective may help refine how cGAS-related stress signaling is understood in targeted therapy resistance.

Several limitations should be acknowledged. Functional homologous recombination (HR)/non-homologous end joining (NHEJ) assays were not performed, and downstream canonical cGAS-STING markers were not directly measured. The precise acquired resistance mechanism of H1975/OR, including T790M status or other resistance-associated alterations, remains to be further defined. The xenograft experiments did not directly evaluate *in vivo* EGFR-TKI resensitization. In addition, the biochemical mechanism by which cGAS influences p-Nrf2 signaling, as well as the proposed cGAS–Nrf2 interface, requires further validation through kinase/phosphatase studies and biophysical binding assays. These limitations indicate that some mechanistic links should remain framed as proposed or inferred models.

In summary, the present study suggests that cGAS downregulation is associated with EGFR-TKI resistance in NSCLC. EGFR-TKI-sensitive cells showed more evident DNA damage, cytosolic dsDNA accumulation, cGAS induction, and ferroptosis-related staining/biochemical changes, whereas resistant cells displayed a comparatively attenuated DNA damage/cGAS response and a more ferroptosis-resistant redox state. Within this setting, cGAS may influence ferroptosis susceptibility through a p-Nrf2–SIRT3-related regulatory axis (Fig. 7). This framework may provide a basis for future studies exploring cGAS-associated redox adaptation as a context-dependent vulnerability in EGFR-TKI-resistant NSCLC.

## 5 Conclusion

The present study suggests that reduced cGAS expression and attenuated ferroptosis-associated oxidative damage may be characteristic of EGFR-TKI-resistant NSCLC cells. Compared with their parental counterparts, resistant cells exhibited less pronounced EGFR-TKI-induced DNA damage, cytosolic dsDNA accumulation, cGAS induction, and ferroptosis-related changes. cGAS knockdown was associated with enhanced malignant phenotypes and a redox profile indicative of reduced susceptibility to ferroptotic injury, whereas cGAS overexpression in resistant cells produced largely opposite effects. Moreover, cGAS modulation was accompanied by changes in Nrf2 Ser40 phosphorylation, Nrf2 subcellular distribution, and SIRT3 expression and activity. The partial reversal of these changes following Nrf2 or SIRT3 manipulation further suggests that the p-Nrf2/SIRT3 pathway may contribute to cGAS-associated redox regulation. Taken together, these findings support a potential role for reduced cGAS expression in maintaining antioxidant capacity and limiting ferroptotic damage in EGFR-TKI-resistant NSCLC. Further studies using patient-derived specimens and clinically relevant models are warranted to clarify the underlying mechanisms and evaluate the potential translational relevance of these findings.

## Data Availability

All data generated or analyzed during this study are included in this published article and are available from the corresponding author upon reasonable request. The publicly available transcriptomic datasets analyzed in this study were obtained from the National Center for Biotechnology Information Gene Expression Omnibus (NCBI GEO) database. GSE172002, entitled “Differential mRNA expression analysis of EGFR-TKI-sensitive cells vs. EGFR-TKI-resistant cells,” is available at https://www.ncbi.nlm.nih.gov/geo/query/acc.cgi?acc=GSE172002. GSE236654, entitled “Disclosing potential therapeutic targets associated with Osimertinib resistance in non-small cell lung cancer cell line H1975,” is available at https://www.ncbi.nlm.nih.gov/geo/query/acc.cgi?acc=GSE236654. All other data generated or analyzed during this study are included in this published article and its supplementary information files. Additional information is available from the corresponding author upon reasonable request.

## Supplementary Materials

The supplementary material is available online at https://www.techscience.com/doi/10.32604/or.2026.082400/s1.

## Abbreviations

The following abbreviations are used in this manuscript:

ACSL4	Acyl-CoA synthetase long-chain family member 4
AOD	Average optical density
AKT	Protein kinase B
BCA	Bicinchoninic acid assay
BRCA1	Breast cancer type 1 susceptibility protein
BSA	Bovine serum albumin
CCK-8	Cell Counting Kit-8
cGAMP	Cyclic GMP–AMP
cGAS	Cyclic GMP–AMP synthase
Co-IP	Co-immunoprecipitation
DAB	3,3′-Diaminobenzidine
DAPI	4′,6-Diamidino-2-phenylindole
DDR	DNA damage response
DEG	Differentially expressed gene
DMEM	Dulbecco’s modified Eagle medium
DMSO	Dimethyl sulfoxide
DNA-PK	DNA-dependent protein kinase
DSB	Double-strand break
dsDNA	Double-stranded DNA
EdU	5-Ethynyl-2′-deoxyuridine
EGFR	Epidermal growth factor receptor
EGFR-TKI	Epidermal growth factor receptor tyrosine kinase inhibitor
FBS	Fetal bovine serum
FDR	False discovery rate
Fe^2+^	Ferrous ion
Fer-1	Ferrostatin-1
GEO	Gene Expression Omnibus
GPX4	Glutathione peroxidase 4
GSEA	Gene set enrichment analysis
GSH	Glutathione
GSCA	Gene Set Cancer Analysis
HADDOCK	High Ambiguity Driven biomolecular DOCKing
HR	Homologous recombination
IC_50_	Half-maximal inhibitory concentration
IF	Immunofluorescence
IHC	Immunohistochemistry
LUAD	Lung adenocarcinoma
LUSC	Lung squamous cell carcinoma
MDA	Malondialdehyde
MMP-9	Matrix metalloproteinase 9
MSigDB	Molecular Signatures Database
mtDNA	Mitochondrial DNA
mito-ROS	Mitochondrial reactive oxygen species
NES	Normalized enrichment score
NFE2L2	Nuclear factor erythroid 2-like 2 gene
NHEJ	Non-homologous end joining
Nrf2	Nuclear factor erythroid 2-related factor 2
NSCLC	Non-small cell lung cancer
PBS	Phosphate-buffered saline
pLDDT	Predicted local distance difference test
qRT-PCR	Quantitative reverse transcription polymerase chain reaction
ROS	Reactive oxygen species
SIRT3	Sirtuin 3
SLC7A11	Solute carrier family 7 member 11
STING	Stimulator of interferon genes
TEM	Transmission electron microscopy
WB	Western blotting
WP4655	WikiPathways Cytosolic DNA-sensing pathway
xCT	Cystine/glutamate antiporter light chain
γ-H2AX	Phosphorylated histone H2AX
