# Supplementary material for: cGAS Downregulation Contributes to EGFR-TKI Resistance in NSCLC through the p-Nrf2–SIRT3–ROS/Ferroptosis Axis
Source: Oncol Res. 2026 Sep 14;34(10):17. doi: 10.32604/or.2026.082400 (PMC13591339; doi:10.32604/or.2026.082400)
Supplement: Supplementary file 1 [file OncolRes-34-82400-s001.zip › Supplementary_Figures_S1-S5.docx]

**Figure S1. EGFR-TKI-resistant NSCLC cells exhibit reduced inhibition of EGFR downstream signaling after drug treatment.** (A) Representative western blot images and densitometric quantification of p-EGFR, EGFR, p-AKT1, AKT1, p-ERK1/2, and ERK1/2 protein levels in H1975 and osimertinib-resistant H1975/OR cells treated with vehicle or osimertinib. (B) Representative western blot images and quantification of these signaling proteins in PC-9 and gefitinib-resistant PC-9/GR cells treated with vehicle or gefitinib. β-actin was used as the loading control. Data are presented as mean ± SD. **p* < 0.05, ***p* < 0.01; *ns*, not significant.

**Figure S2. EGFR-TKI treatment induces mitochondrial dysfunction and ferroptosis-associated oxidative damage in sensitive NSCLC cells, which is attenuated by Ferrostatin-1.** (A) Representative JC-1 staining images and quantification of the JC-1 red/green fluorescence intensity ratio in H1975 and H1975/OR cells treated with osimertinib, and in PC-9 and PC-9/GR cells treated with gefitinib for 0 or 24 h. JC-1 aggregates are shown in red, JC-1 monomers in green, and nuclei were counterstained with Hoechst. (B) Quantification of intracellular GSH and MDA levels in parental and EGFR-TKI-resistant cells after osimertinib or gefitinib treatment for 0 or 24 h. (C) Representative fluorescence images showing ROS-associated fluorescence signals in PC-9 cells treated with gefitinib and in H1975 cells treated with osimertinib, in the presence or absence of the Fer-1. (D) Quantification of GSH levels in PC-9 and H1975 cells following EGFR-TKI treatment with or without Fer-1. (E) Quantification of MDA levels in PC-9 and H1975 cells following EGFR-TKI treatment with or without Fer-1. Scale bar = 200 μm. Data are presented as mean ± SD. **p* < 0.05, ***p* < 0.01, ****p* < 0.001; *ns*, not significant.

**Figure S3. cGAS regulates GSH and MDA levels in xenograft tumor tissues.** (A) Quantification of GSH levels in tumor tissues derived from H1975, H1975/OR, PC-9, and PC-9/GR xenografts following cGAS knockdown or overexpression. (B) Quantification of MDA levels in the corresponding xenograft tumor tissues. cGAS knockdown increased GSH levels and reduced MDA accumulation in parental cells, whereas cGAS overexpression decreased GSH levels and increased MDA accumulation in EGFR-TKI-resistant cells. Data are presented as mean ± SD. **p* < 0.05, ***p* < 0.01; *ns*, not significant.

**Figure S4. Ferrostatin-1 attenuates cGAS overexpression-induced ferroptosis-associated oxidative damage in EGFR-TKI-resistant NSCLC cells.** (A) Representative FerroOrange staining images showing intracellular Fe²⁺-associated fluorescence in PC-9/GR and H1975/OR cells treated with gefitinib or osimertinib, respectively, under cGAS overexpression with or without Fer-1. Scale bar = 200 μm. (B) Representative C11 BODIPY 581/591 staining images showing lipid peroxidation, with non-oxidized lipids shown in red and oxidized lipids shown in green. Scale bar = 200 μm. (C) Representative fluorescence images showing intracellular ROS-associated fluorescence signals in the indicated treatment groups. Scale bar = 200 μm. (D) Quantification of GSH levels in PC-9/GR and H1975/OR cells under the indicated treatments. (E) Quantification of MDA levels in PC-9/GR and H1975/OR cells under the indicated treatments. Hoechst was used for nuclear counterstaining. Data are presented as mean ± SD. **p* < 0.05.

**Figure S5. cGAS regulates SIRT3 expression and activity through the Nrf2/SIRT3 axis in EGFR-TKI-sensitive and -resistant NSCLC cells**. (A) Relative SIRT3 mRNA expression in H1975 and PC-9 cells following cGAS knockdown, and in H1975/OR and PC-9/GR cells following cGAS overexpression. (B) SIRT3 activity in H1975 and PC-9 cells following cGAS knockdown, and in H1975/OR and PC-9/GR cells following cGAS overexpression. (C) Predicted structural model of cGAS and Nrf2. (D) Molecular docking analysis of cGAS and Nrf2. (E) Relative cGAS mRNA expression after SIRT3 overexpression or knockdown. (F) Quantification of cGAS, Nrf2, p-Nrf2, and SIRT3 protein levels after Nrf2 overexpression or knockdown. (G) Relative SIRT3 mRNA expression following Nrf2 overexpression or knockdown. (H) SIRT3 activity following Nrf2 overexpression or knockdown. (I) Quantification of cytoplasmic Nrf2 and p-Nrf2 levels under combined cGAS and SIRT3 modulation in H1975/OR and PC-9/GR cells. (J) Quantification of nuclear Nrf2 and p-Nrf2 levels under combined cGAS and SIRT3 modulation in H1975/OR and PC-9/GR cells. Data are presented as mean ± SD. **p* < 0.05, ***p* < 0.01, ****p* < 0.001; *ns*, not significant.
